# Modelling time-varying interactions in complex systems: the Score Driven Kinetic Ising Model

**DOI:** 10.1038/s41598-022-23770-0

**Published:** 2022-11-11

**Authors:** Carlo Campajola, Domenico Di Gangi, Fabrizio Lillo, Daniele Tantari

**Affiliations:** 1 Scuola Normale Superiore, p. dei Cavalieri 7, 56126 Pisa, Italy; 2 UZH Blockchain Center, University of Zürich, Rämistrasse 71, 8006 Zürich, Switzerland; 3 Department of Informatics, University of Zürich, Andreasstrasse 15, 8050 Zürich, Switzerland; 4 Institute of Information Science and Technologies, National Research Council (CNR), via G. Moruzzi 1, 56124 Pisa, Italy; 5 Department of Mathematics, University of Bologna, p. di Porta San Donato 5, 40126 Bologna, Italy

**Keywords:** Applied mathematics, Statistical physics, thermodynamics and nonlinear dynamics

## Abstract

A common issue when analyzing real-world complex systems is that the interactions between their elements often change over time. Here we propose a new modeling approach for time-varying interactions generalising the well-known Kinetic Ising Model, a minimalistic pairwise constant interactions model which has found applications in several scientific disciplines. Keeping arbitrary choices of dynamics to a minimum and seeking information theoretical optimality, the Score-Driven methodology allows to extract from data and interpret the presence of temporal patterns describing time-varying interactions. We identify a parameter whose value at a given time can be directly associated with the local predictability of the dynamics and we introduce a method to dynamically learn its value from the data, without specifying parametrically the system’s dynamics. We extend our framework to disentangle different sources (e.g. endogenous vs exogenous) of predictability in real time, and show how our methodology applies to a variety of complex systems such as financial markets, temporal (social) networks, and neuronal populations.

## Introduction

Complex systems, characterized by a large number of simple components that interact with each other, have been an increasingly important field of study over the last decades. Interactions make the whole more than the sum of its parts: for this reason the effort when modeling complex systems is ultimately directed to understand how interactions arise, how to parametrize them into quantitative models and how to estimate them from empirical measurements.

One complication that is ubiquitous to real complex systems, but very rarely considered in modeling, is that interactions change over time: traders in financial markets continuously adapt their strategic decision-making to new information^1,2^, neurons reinforce (or inhibit) connections in response to stimuli^3^. As we show in this work, a modeling approach assuming that all the interactions are constant can sometimes lead to spurious estimations, which can be avoided only with very strong limitations to sample selection and experimental design.

In this article we propose a novel approach to the development of models for time-varying interactions based on the generalization of a minimalistic constant-interactions model, commonly used in many scientific disciplines: the Kinetic Ising Model (KIM)^4–6^. We show that this generalization allows to describe conditions where the predictability of the observed process is variable, while commonly employed constant interaction models fail in this respect. More importantly, our modeling approach does not assume that the causes or the dynamics of the variable interactions are known, but they are estimated (or filtered) from the data themselves. Thus, different types of time-varying interactions can be present in the investigated system, including non-stationarities of various form (regime-shift, seasonalities, etc.). Indeed it often occurs that the modeler has no insight on the nature of the underlying dynamics of interactions: the dynamics that is given to the time-varying parameters then needs to be as agnostic as possible with respect to the actual generating dynamics, *i.e.* be robust to model misspecification errors.

The focus of the paper is the score-driven generalization of the KIM, which is the dynamical counterpart of the celebrated Ising spin glass model^7,8^. Ising models in general are known to be among the simplest models of complex systems that have been developed in the field of statistical physics and are at the roots of the theory on collective behavior and phase transitions. Their popularity is also due to the fact that they fall into the class of Maximum Entropy models^9–12^ when only average values and cross correlations are taken into account. The KIM in particular has been adopted in a variety of fields, such as neuroscience^13,14^, computational biology^15,16^, economics and finance^17–20^ and has been studied in the literature of machine learning^21–23^ to understand recurrent neural network models.

The KIM describes the time evolution of a set of *N* binary variables $s(t) \in \lbrace -1, 1 \rbrace ^N$ for $t = 1, \dots , T$, typically called “spins”, which can influence each other through a time lagged interaction. It involves two main parameters: a $N \times N$ interaction matrix *J* and a *N*-dimensional vector *h* of spin-specific biases, which we summarize as $\Theta = (J,h)$. The model is Markovian with synchronous dynamics, characterized by the transition probability

1 $$\begin{aligned} p(s(t) \vert s(t-1); \beta , \Theta ) = \frac{e^{\beta \sum _i s_i(t) g_i(t)} }{Z(t)} \end{aligned}$$

where *Z*(*t*) is a normalizing constant and $\beta$ is a parameter that determines the overall strength of interactions between spins, known as the *inverse temperature*. The quantity $g_i(t)\equiv \sum _{j=1}^N J_{ij} s_j(t-1) + h_i$ is called the *effective field* perceived by spin *i* at time *t*. Furthermore, it is possible in principle to introduce dependency on *K* external covariates $x_k(t)$, by adding a term $b_{ik}x_k(t)$ to $g_i(t)$ for each $k \in \lbrace 1, \dots , K \rbrace$^20^.

In the standard KIM the interactions *J* are constant in time. Let us introduce our framework by considering a sequence of matrices *J*(*t*), such that $J_{ij}(t)$ represents the strength of interaction between the spins $s_i(t)$ and $s_j(t-1)$, and assume the value of *J*(*t*) is evolving in time adapting to the current state vector, i.e.

2 $$\begin{aligned} J_{ij} (t+1) = F (J(t),s(t)). \end{aligned}$$

The updating functional *F* is determined by general assumptions based on information theory principles. First of all, one can assume that interactions vary based on surprise: the more an observation of the system’s state is “unexpected”, the more the relations between its components will change. In social systems, for example, friendship relationships can get damaged if not constantly fed or may arise from unexpected gestures of openness. This is also a common principle in biological learning processes and artificial neural networks, where the least expected inputs have the largest impact on the values of the synapses or inter-units weights^24^. The most common measure of surprise is minus the logarithm of the conditional likelihood of observing the current state given the current level of interactions, $- \log p(s(t) \vert J(t))$. As a second principle we assume that the system’s reaction to surprise is to adapt to it, making what had been unexpected at that moment less surprising in the future. This implies that the interactions change to reduce surprise, i.e. increase the log likelihood of the last observation: the next value will be a linear combination of the present value and a contribution in the direction of maximum likelihood, that is

3 $$\begin{aligned} J(t+1) = w + B J(t) + A(t) \frac{\partial \log p (s(t) \vert J(t))}{\partial J(t)}, \end{aligned}$$

Following from the above argument, Eq. 3 is the combination of an *autoregressive* part $w + BJ(t)$ with a *gradient ascent* part $A(t) \frac{\partial \log p (s(t) \vert J(t))}{\partial J(t)}$ driving the dynamics. *w* and *B* are constant parameters that govern the mean value of *J*(*t*) and its persistence, while *A*(*t*) is a (possibly time-dependent) learning rate parameter quantifying how reactive are the interactions to the realized state of the system.

This type of *observation-driven*^25^ dynamics has been recently formalized, defining the class of *score-driven models*^26,27^. These have been shown to be an optimal choice among observation-driven models when minimizing the Kullback-Leibler divergence to an unknown generating probability distribution^28^ and have risen in popularity in econometrics^29^ as well as network science^30,31^. For a generic multivariate time series of length *T*, $\lbrace s(t) \rbrace _{t=1}^T$ where $s(t) \in \mathbb {R}^N$, and a model with conditional log-likelihood $\mathscr{L}(t) = \log p (s(t) \vert S(t-1), f(t) )$ depending on a vector of time-varying parameters $f(t) \in \mathbb {R}^M$ and past observations $S(t-1) = \lbrace s(k) \rbrace _{k = 1}^{t-1}$, a score-driven model assumes that the time evolution of *f*(*t*) is ruled by the recursive relation

4 $$\begin{aligned} f(t+1) = w + B f(t) + A \mathscr{I}^{-1/2}(t) \frac{\partial \mathscr{L}(t)}{\partial f(t)} \end{aligned}$$

where *w*, *B* and *A* are a set of static parameters. In the rest of the article we will call $\nabla _t = \frac{\partial \mathscr{L}(t)}{\partial f(t)}$ the *score function* at time *t*, hence the name *score-driven model*. $\mathscr{I}^{-1/2}(t)$ is a $M\times M$ matrix regularizing the convexity, that we choose to be the inverse of the square root of the Fisher information associated with $\mathscr{L}(t)$, thus letting the last term of Eq. 4 be a random variable with unit variance and zero mean by definition.

As is clear from Eq. 4, the score $\nabla _t$ drives the time evolution of *f*(*t*) and no additional source of noise is introduced. This means that, given $\mathscr{L}(t)$, any new observation produces a deterministic update of the time-varying parameters. The update can remind the reader of a Newton-like method for optimization, in that the parameters are moved towards the maximum of the likelihood at each realization of the observations while keeping memory of the time evolution through the *B* static parameter.

The fact that time-varying parameters are deterministic functions of the observations has some intrinsic advantages also for estimation, as the elimination of unobservable noise removes the necessity of implementing computationally intensive Monte Carlo simulations to calculate the model likelihood. Furthermore, an observation-driven model can be used as a filter: having knowledge of all the static parameters (*e.g.* because they were previously estimated on a training set), the time-varying parameters can be updated with no effort every time a new data point is observed. In the following we will make wide use of the score-driven model as filter of an unknown dynamics and in the Supplementary Information (SI) we provide more context for it by revisiting the simple case of a GARCH process^32^.

As in Eq. (3) for the interactions, we propose to use Eq. (4) to provide a dynamics to the parameters $(\beta ,\Theta )$ thus introducing a class of Score Driven generalizations of the KIM. Notice however that the number of parameters in the KIM is large, $O(N^2)$: as customary in high-dimensional modeling, in the following we will propose two parsimonious and informed parameter restrictions that simplify the treatment and define two kinds of Score-Driven KIM, each tailored to highlight different effects.

As we show in this article, the development of a score-driven KIM addresses three important points: first, introducing a dynamical noise parameter $\beta (t)$ allows to gain real time insight on the ability of the model to explain the observed dynamics, thus leading to more informed forecasts; second, neglecting time variability of parameters by estimating a standard KIM turns out to produce systematic errors, in particular the estimated values are different from the time-averaged values that generated the sample; third, by introducing a convenient factorization for the model parameters, it is possible to discriminate whether an observation is better explained by endogenous interactions with other variables or by exogenous effects, offering an improved understanding of the dynamics that generated the data even when these effects are not constant over time. We prove the effectiveness of our modeling approach by extensive numerical simulations and by empirical application to different complex systems.

## Results

### The Dynamical Noise KIM

The first score-driven KIM we propose addresses the first two points made above: the real time prediction of forecast accuracy and the correction of systematic estimation errors of a constant parameter model.

**Figure 1:**
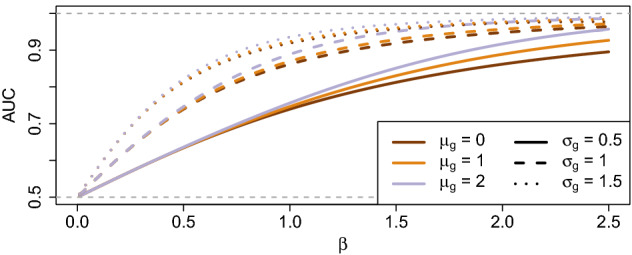
Theoretical AUC as a function of $\beta$ assuming $g_i$ is Gaussian distributed with mean $\mu _g$ and standard deviation $\sigma _g$. Different colors correspond to different values of $\mu _g$, while line types identify values of $\sigma _g$. We see that increasing $\beta$ has the effect of reducing the uncertainty on the random variable $s_i(t)$, keeping $g_i$ unchanged. Grey dashed lines at $\textrm{AUC}=0.5$ and $\textrm{AUC}=1$ are guides to the eye.

In general, at a given time $t-1$ with an observation $s(t-1)$, it is possible to use the KIM to produce one-step ahead forecasts for *s*(*t*), which we call $\hat{s}_i(t)$. These are obtained as

5 $$\begin{aligned} \hat{s}_i(t) = \textrm{sign} \left[ p \left(s_i(t) = 1 \big \vert s(t-1), \Theta , \beta \right) - \alpha \right] \end{aligned}$$

where $\alpha$ is the threshold level the modeler is willing to use to predict that $s(t)=+1$, and the transition probability is that of Eq. 1. Sweeping the value of $\alpha$ between 0 and 1 one obtains a ROC curve, which in turn can be used to calculate the Area Under the Curve (AUC)^33^, a standard measure for the quality of forecasts. In a nutshell, the AUC has a value of 1 for a perfect predictor and of 0.5 for a completely uninformed one. Figure 1 displays the value of the expected AUC in the KIM as a function of $\beta$ (see also the SI for a detailed analytical derivation), with the assumptions that the $g_i(t)$ are Gaussian distributed with mean $\mu _g$ and standard deviation $\sigma _g$. This is a standard case in the literature and occurs if the $J_{ij}$ entries are Gaussian distributed with zero mean^34^. We see that the AUC is monotonically increasing with $\beta$, and the distribution of the effective fields affects the slope with which the curve converges towards 1. The key message from Fig. 1 though is that the larger is $\beta$ the more reliable is the prediction of the model: if one is able to estimate $\beta$ they can assess in real time how accurate the model is in forecasting the next observation.

However, it is known that given a set of observations the parameter $\beta$ in the KIM of Eq. 1 is not identifiable^35^, i.e cannot be estimated. In fact, for any two values $\beta _1$ and $\beta _2$ there are also two sets of parameters $\Theta _1$ and $\Theta _2$ such that $p(s(t) \vert s(t-1);\beta _1, \Theta _1) = p(s(t) \vert s(t-1); \beta _2, \Theta _2)$ for all *s*(*t*). For this reason in inference problems it is typically assumed that $\beta =1$, incorporating its effect in the other parameters.

We overcome this limitation by introducing the Dynamical Noise KIM (DyNoKIM), defined by letting $\beta$ in Eq. 1 be time-varying with score-driven dynamics, while all other parameters remain constant. The DyNoKIM then follows the transition probability

6 $$\begin{aligned} p(s(t) \vert s(t-1); J, \beta (t)) = \frac{ e^{ \beta (t) \sum _{i} s_i(t) g_i(t)}}{Z(t) } \end{aligned}$$

with $Z(t) = \prod _i 2 \cosh \left[ \beta (t) g_i(t)\right]$. We give score-driven dynamics to $f(t) = \log \beta (t)$, as $\beta$ has to be positive:

7 $$\begin{aligned} \log \beta (t+1) = w + B \log \beta (t) + A \mathscr{I}^{-1/2}(t) \nabla _t \end{aligned}$$

where *w*, *B* and *A* are scalar parameters, $\mathscr{I}(t)$ is the Fisher Information and $\nabla _t = \frac{\partial \log p(s(t) \vert s(t-1), \beta (t))}{\partial \beta (t)}$ is the score.

When $\beta$ is made time-varying, the identification problem is limited to its time average value $\langle \beta \rangle$ (which still needs to be assumed equal to 1), while its local value can be inferred from the data. This simple extension to the KIM then turns out to be extremely useful: as it is now possible to estimate $\beta (t)$, one can measure in real time the reliability of the next forecast by considering the expected AUC of Fig.  1. Notice that the prediction is made using Eq.  5, once $\beta$ is replaced with the $\beta (t)$ of the DyNoKIM, and thus it is fully causal since $\beta (t)$ depends only from past observations $S(t-1)$ through Eq. 7.

The parameters of Eq. 7 are inferred by Maximum Likelihood Estimation (see “Methods”). We numerically find that the model parameters can be consistently estimated and report a detailed analysis in the SI.

**Figure 2:**
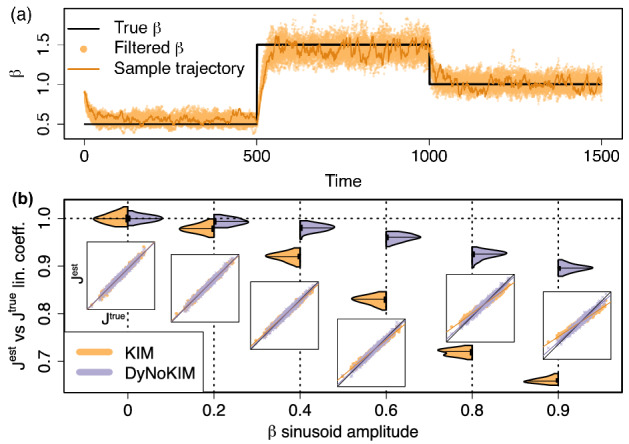
(**a**) Estimation of $\beta$ from simulated time series of spins, generated with a KIM with piecewise constant $\beta$ (black line). The $\beta (t)$ estimated with the DyNoKIM (yellow dots) over 30 simulations is consistently close to the true value. A sample estimated trajectory is shown in darker color. (**b**) Estimation of *J* under model misspecification. We simulate 60 time series generated with a KIM and $\beta (t) = 1 + \mathcal {K} \sin (\omega t)$, $\omega = 2\pi /300$, $T=3000$, $N=30$, $J_{ij} \sim \mathcal {N}(0, 1/\sqrt{N})$, $h_i = 0 \; \forall \, i$, then estimate the DyNoKIM. The main panel shows the distribution of the coefficient of a linear regression between the estimated and true *J* values, using the KIM and a DyNoKIM. Insets show example scatter plots of the true *J* values (*x* axis) and the estimated values (*y* axis) using the standard KIM (yellow points) or the DyNoKIM (purple crosses).

We study the model’s ability to retrieve a temporal pattern $\beta (t)$ also when the data generating process is not score-driven. Indeed there is little reason to believe that this sort of dynamics is an actual data generating process for real-world complex systems, where $\beta$ might follow exogenous and unknown dynamics. The power of score-driven models lies also in the capability of estimating time-varying parameters without actually requiring any assumption on their true dynamical laws, behaving as filters for their underlying unknown dynamics. To show this we simulate 30 time series of length $T=1500$ using a KIM where $\beta$ is set to 0.5, 1.5 and 1.0 for 500 time steps each, and then feed the resulting *s*(*t*) to the inference algorithm of the DyNoKIM to estimate the static parameters and $\beta (t)$. In Fig. 2a we show the “true”$\beta$ used for the simulation and compare it with the ones filtered by the DyNoKIM: even if the time series are not generated using the score-driven model, the DyNoKIM is capable of estimating the true $\beta$ with remarkable accuracy.

One could argue that the estimated parameters of the DyNoKIM are equivalent to those of a standard KIM with a constant $\beta$ equal to its time average $\langle \beta \rangle$. This is not the case. Fig. 2b shows the results for a set of simulations where $\beta (t)$ follows a deterministic sinusoidal dynamics, $\beta (t) = 1 + \mathcal {K} \sin \omega t$, varying the amplitude $\mathcal {K}$, and the time evolution of *s*(*t*) is given by Eq. 6. For each value of $\mathcal {K}$ we simulate 60 time series of length *T* and fit both the KIM and the DyNoKIM; we then compare the estimated matrix of interactions $J^{est}$ with the one that was used to generate the data, $J^{true}$, by fitting a linear regression $J^{est}_{ij} = a + bJ^{true}_{ij} + \varepsilon$. We see from Fig. 2b that when $\beta$ is not constant, the KIM underestimates the absolute value of the parameters, highlighted by the fact that $b < 1$ (and $a \approx 0$, not shown). The error is greatly reduced in the DyNoKIM thanks to the way in which we solve the indetermination of $\langle \beta \rangle$: after the model parameters are estimated and a filtered $\beta ^{est}(t)$ is found, we normalize its mean to 1 and multiply the estimated $J^{est}$ by the same factor, leaving the likelihood of the model unchanged. This result supports our argument that using a KIM on data where parameters of the data generating process are time varying can be misleading and leads to significant errors, something that can be overcome by adopting a score driven model.

**Figure 3:**
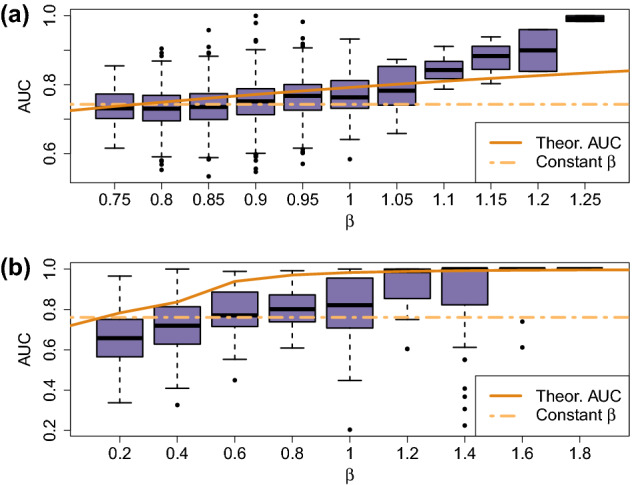
AUC statistics compared to $\beta (t)$ in applications of DyNoKIM. (**a**) AUC values for stock price activity on November 19, 2019 aggregated for different values of $\beta (t)$, compared to the theoretical expected AUC with Gaussian $g_i(t)$ and to the performance with constant $\beta$; (**b**) AUC values for link prediction in the SocioPatterns dataset, compared with the theoretical expected AUC and the constant $\beta$ benchmark.

### Forecasting stock price activity with DyNoKIM

We apply the concepts above to a simple setting, where the KIM is very likely misspecified, yet it can produce real-time forecasts. Measuring high-frequency price volatility in financial markets is a non-trivial task that has been at the core of research in quantitative finance over the last two decades^36^. Volatility is in fact a latent process which is hard to measure for reasons that range from price staleness to microstructural effects like price discretization and bid-ask bounce. Price activity, namely the binary time series marking the events of price changes - regardless of direction and size - is a proxy for high-frequency volatility that has been recently used to quantify the endogeneity in the price formation^37–41^.

Indeed, at high-frequency, price movements are often of the minimum possible size, called a *tick* - which for US equities is generally 0.01 Dollars. This means that even a binary variable that marks whether the price has changed or not captures a significant part of the information about volatility, as the amount of variation is not particularly heterogeneous.

We then test the DyNoKIM as a tool to measure the predictability of stock price activity at high frequency. The advantage with respect to standard methods is twofold: first, we are able to model the dynamics of a large panel of assets, hence considering volatility spillovers between them; second, the score driven approach allows us to measure the local predictability of price activity in real time. We study the 100 largest capitalization stocks in the US markets NASDAQ and NYSE over 11 trading days. Price activity is defined as a binary variable $s_i(t)$ for each stock *i*, taking value $+1$ if the stock price has changed in the interval $(t-1,t]$ and $-1$ otherwise, with time discretized at 5 seconds. We focus our attention on the lagged interdependencies among different stocks, by applying the DyNoKIM to the multivariate time series *s*(*t*).

Our theoretical results from Fig. 1 suggest to use $\beta$ to quantify the reliability of forecasts of price activity using this model. We estimate the model static parameters $\Theta , w, B$ and *A* on daily sub-samples, and use the estimates on the following day to filter the values of $\beta (t)$. We use Eq. 5 to produce forecasts and measure their accuracy using the AUC metric. To ensure that there is reason to model the system with time-varying $\beta$, we apply a Lagrange Multiplier test^42^ with a null hypothesis of constant $\beta$, finding strong rejections of the null at the $p<0.001$ level for every day in the sample. Further information about this test can be found in the “Methods” section.

We show our results in Fig. 3a. We empirically observe that when the filtered value of $\beta (t)$ is large, the following forecast of activity $\hat{s}(t)$ is systematically more reliable, as the AUC is larger. We find a good agreement between the empirical results and the theoretical values shown in Fig. 1 under the assumption of Gaussian effective fields $g_i$, where some discrepancy is easily explained by the non-Gaussianity of the estimated *J*. Thus we conclude that the DyNoKIM can be effectively used to model high frequency volatility of a large portfolio of stocks and to measure in real time its level of predictability.

### Link prediction in temporal networks with DyNoKIM

In our second application, we show that DyNoKIM can be used to model temporal networks. In particular we show that DyNoKIM dynamically provides the level of predictability of links of the network by exploiting again the relation between $\beta (t)$ and AUC.

Networks are a paradigmatic tool to describe pairwise relations in complex systems^43,44^ and applications include human mobility^45^, migration^46^, disease spreading^47^, international trade^48^ and financial stability^49^, to mention a few. More recently, the increasing availability of time varying relational data stimulated a widespread and fast growing interest in the analysis of temporal networks^50^. It also motivated the development of a number of models to describe the dynamics of temporal networks^51,52^.

A network, defined by a set of *M* nodes and a set of links between pairs of nodes, can be described by an $M\times M$ binary adjacency matrix $G \in \lbrace 0, 1 \rbrace ^{M\times M}$, where $G_{ij} = 1$ if a link between nodes *i* and *j* is present and $G_{ij} = 0$ otherwise. When the relation described by the links is not directional, $G_{ij} = G_{ji}$ and the network is said to be undirected. We consider temporal networks where the number of nodes *M* is fixed across multiple time steps and indicate the adjacency matrix of the graph at time *t* by $G\left( t \right)$.

In order to use the KIM to model a temporal network, we map the elements of the adjacency matrix into spins, associating a present link to a spin $+1$ and an absent link to a spin $-1$. In this way we represent each adjacency matrix $G\left( t \right)$ as a vector $s(t) \in \lbrace -1, 1 \rbrace ^N$ where $N = M(M-1)/2$, assuming the network to be undirected and without self loops. In light of this mapping, the matrix *J* now captures the tendency of links to influence each other at lag one - for example the diagonal terms can be interpreted as measuring link persistence, while the elements of *h* are associated with the idiosyncratic probability to observe a given link.

Interestingly, such a mapping highlights that the (standard) KIM can be seen as belonging to the Temporal Exponential Random Graph Model^52^ family, as we discuss in the SI. Moreover, it turns out that a large subset of possible specifications from this family can be mapped into a KIM. Hence, the score driven KIM that we propose here is an extension of the Temporal Exponential Random Graph Model allowing its parameters to evolve in time. This frames DyNoKIM also as a contribution to the literature on network models with time varying parameters, alongside with a recent extension of a different, but related, family called Exponential Random Graphs^53^ to its score driven version^30^.

We apply DyNoKIM to forecast the presence of a link at time $t+1$ given the observations available up to time *t* for a real world temporal network describing close proximity between workers at the Institut National de Veille Sanitaire in Saint-Maurice^54^. The data was collected with the sensing platform developed by the SocioPatterns collaboration and describea situations of face-to-face proximity between pairs of workers lasting at least 20 seconds. The observations cover 10 working days, from June 24 to July 3, 2013. For each day, we construct the time series of adjacency matrices, at a frequency of 20 seconds between 7:30 am and 5:30 pm. A link between two workers is present if they face each other at a distance less than 1.5 meters and is absent otherwise. As is often the case in real temporal networks, a large number of links is never, or very rarely, observed. Since for such trivial links the prediction problem is not interesting, and to keep the computational complexity to a reasonable level, we consider only the subset of the 100 most active links in each day. For each day, we estimate the DyNoKIM on a training set consisting of the first $75\%$ of observations and then use the remaining $25\%$ for out of sample validation. For each *t* we compute the AUC and report in Fig. 3b the aggregated results for all days. As in the financial application, we observe a monotonically increasing relation between $\beta (t)$ and AUC, indicating that DyNoKIM is a reliable tool to dynamically quantify forecast accuracy also in applications to temporal networks data. Also in this case, we observe a good agreement with the theoretical prediction, with differences explainable by the non Gaussianity of the estimated matrix *J*.

These two empirical examples show that our theoretical results for the DyNoKIM are indeed verified in realistic applications and that using this method - which we believe could be applied even to more sophisticated models - can result in a significant gain in the use of forecasting models, giving a simple criterion to discriminate when to trust (or not) the forecasts.

### The Dynamic Endogeneity KIM

In this last section we explore a more general specification of score-driven KIM, the Dynamic Endogeneity Kinetic Ising Model (DyEnKIM), where we assume that the parameters *J* and *h* have a specific time-varying factorization. Going back to Eq. 1, we now impose the following structure to the parameters:

8 $$\begin{aligned} \beta&= 1 \nonumber \\ J_{ij}(t)&= \beta _{diag}(t) J_{ij} \delta _{ij} + \beta _{off}(t) J_{ij} (1 - \delta _{ij}) \nonumber \\ h_i(t)&= \beta _h(t) (h_i + h_0(t)) \end{aligned}$$

where $\delta _{ij}$ is the Kronecker symbol which is 1 if $i=j$ and 0 otherwise.

With this choice we want to be able to discriminate between different components of the observed system dynamics: one associated with the idiosyncratic properties of variable *i* ($\beta _h$), with general trends ($h_0$), with autocorrelations ($\beta _{diag}$), and finally with lagged cross-correlations among variables ($\beta _{off}$). In this formulation each of these time-varying parameters measures the relative importance of one term over the others in the generation of the data, highlighting periods of higher endogeneity of the dynamics (when correlations have higher importance) rather than periods where the dynamics is more idiosyncratic or exogenously driven. We report a consistency analysis for the DyEnKIM in the SI, where we show that even under model misspecification this approach correctly separates the different components of the dynamics and captures their relative importance.

**Table 1:** Summary statistics of the Kolmogorov-Smirnov distances between the distributions of synchronous spikes and pattern frequency and the ones simulated with the KIM and DyEnKIM, taken on 297 experiments. The DyEnKIM consistently outperforms the KIM at reproducing higher-order quantities. We quantify the improvement as the percentage difference between KS distances $\frac{KS_{KIM} - KS_{DyEnKIM}}{KS_{KIM}}$, which is in favor of DyEnKIM in the vast majority of experiments.

	Synchronous Spikes			Zipf Plot		
KS Distance	KIM	DyEnKIM	Improvement	KIM	DyEnKIM	Improvement
Mean	0.17	0.12	22%	0.12	0.07	33%
Median	0.16	0.12	27%	0.12	0.07	43%
1. Quartile	0.14	0.10	6%	0.09	0.05	17%
3. Quartile	0.18	0.14	43%	0.14	0.09	65%

**Figure 4:**
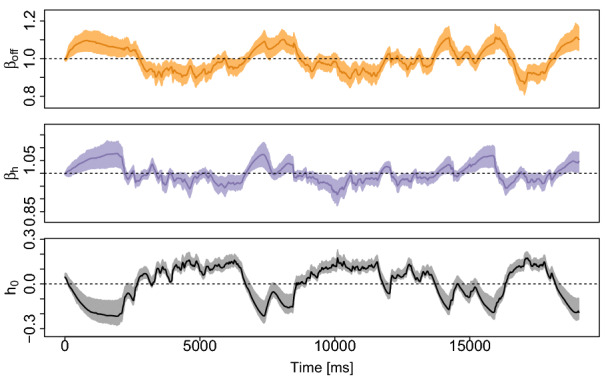
Filtered values of $\beta _{off}$, $\beta _h$, and $h_0(t)$ for salamander retina data, showing the average value across the 297 experiments and the 90% confidence interval (i.e. 268/297 of the filtered values stay within the bands.

### Role of non stationarity in neural data

As an example application of the DyEnKIM, we consider the firing dynamics of a set of neurons. Inferring the network of functional connections between neurons by observing the correlated dynamics of firing has received a lot of attention in the last two decades^10,55^ and the KIM has been extensively used for this purpose^56–58^. The underlying idea is that the (lagged) correlation in the firing of two time series suggests the existence of a physical connection between the two corresponding neurons.

However, as pointed out by Tyrcha et al.^59^, correlated behavior can also be generated by the fact that neurons are subject to a common non-stationary input, for example driven by the external environment. Disentangling the contributions to correlations coming from external drivers and those coming from genuine interactions is critical to reliably identify the network structure between neurons.

To this end Tyrcha et al.^59^ propose an inferential method to achieve this result by considering a KIM with time dependent external fields $h_i(t)$ representing the contribution of the external stimuli and of all the non recorded neurons to the activity of neuron *i* at time *t*. However the inference method requires many “trials”or repetitions of the experiment, under the strong methodological assumption that all the repetitions are obtained under identical conditions, an hypothesis that might be difficult to control in such type of complex experiments.

We now show that DyEnKIM can be used for this purpose *on a single experiment*. We use the data of Tkačik et al.^60^ obtained from a multichannel experiment recording firing patterns of 160 salamander retina neurons, stimulated by a film clip of a swimming fish. The 20s experiment is sampled with time binning of 20ms, where each neuron is associated with a spin $s_i(t)$ taking value $+1$ if the neuron produced a spike in the last time window and $-1$ otherwise. Each experiment contains $T=944$ observations per neuron and we considered the $N=40$ most active neurons. Finally the experiment is repeated 297 times.

We fit the DyEnKIM of Eq. 4 and for each experiment we perform a Lagrange Multiplier test. We find that while for $\beta _{off}(t)$, $\beta _h(t)$, and $h_0(t)$ we reject the null hypothesis of constant parameter in $99.3\%$, $76.8\%$, and $100\%$ of the experiments respectively, this percentage drops to $43.1\%$ for $\beta _{diag}(t)$. For this reason we consider a simplified model where $\beta _{diag}(t)$ is constant, but results are essentially unchanged when considering a time varying $\beta _{diag}(t)$. Fig. 4 shows the filtered temporal dynamics: the solid line is the average value of the parameter at each time across the 297 experiments, while the shaded area is the 90% confidence interval. It is evident that the three parameters show significant variations, likely in response to the external stimulus provided by the film clip and by unobserved neurons.

In order to evaluate how well our model describes the empirical data we consider two statistics: (i) the distribution of the number of synchronous spikes (i.e. the number of $s_i(t) = +1$ within the same time bin) and (ii) the Zipf plot, that is the frequency of each specific spiking pattern *s*(*t*) ranked from most common to most infrequent. Both quantities depend on the many body synchronous correlations among spins, thus are not in principle automatically explained by KIM-type models which fit the pairwise lagged correlations. As a benchmark model we consider a constant parameters KIM estimated on the whole dataset. We show the resulting distributions in the SI. Table 1 quantifies the improvement of the DyEnKIM over the KIM in terms of the Kolmogorov-Smirnov (KS) distance between the empirical distributions and the ones reproduced by simulation of the fitted models. We see that the DyEnKIM produces a simulated distribution which is closer to the empirical one in the vast majority of experiments, and we quantify the improvement from KIM to DyEnKIM by taking the percentage difference between the KS distances. For instance we see that on average the DyEnKIM produces a distribution of synchronous spikes that is 22% closer to the data than the one given by the KIM, and 33% closer when considering the Zipf plot.

It is important to stress once more that, while an approach as in Tyrcha et al.^59^ requires many experiments and the strong methodological assumption that these are identical realizations of the same process, our method to measure time-varying interactions can be performed on a single experiment. Incidentally, one can then use the estimation to test whether the different experiments are statistically equivalent by comparing the estimates across replicas. Moreover our model has only three time dependent scalars, while the model of Tyrcha et al.^59^ requires a time dependent field for each of the *N* neurons, thus being highly parametrized with a modeled dynamics strongly constrained by the data.

In the SI we also report two applications of DyEnKIM to financial data, where we use it to disentangle endogenous from exogenous contributions to stock price activity and trading patterns in correspondence of particular events.

## Discussion

We have applied the score-driven methodology to extend the Kinetic Ising Model to a time-varying parameters formulation, introducing two new models for complex systems: the Dynamical Noise Kinetic Ising Model (DyNoKIM) and the Dynamic Endogeneity Kinetic Ising Model (DyEnKIM). We showed that the DyNoKIM, characterized by a time-varying noise level parameter $\beta (t)$, has a clear utility in forecasting applications, as the Area Under the ROC Curve can be showed to be a growing function of $\beta (t)$, while the DyEnKIM can be used to discriminate between endogenous and exogenous effects in the evolution of a multivariate time series.

We then provided example applications of the two models. We successfully employed the DyNoKIM to quantify the real-time forecasting accuracy of stock price activities in the US stock market, as well as the real-time link prediction accuracy in a temporal social network. The result, largely matching the predictions from theory and simulations, is a methodological breakthrough for the real-world application of time-varying parameter models of complex systems, opening to the possibility of implementing real-time indicators quantifying the accuracy of model-based predictions.

We have then applied the DyEnKIM to model a population of salamander retina neurons. We designed the DyEnKIM to disentangle the effects of interactions from the ones of exogenous sources on the observed collective dynamics, a task that is typically non-trivial but nonetheless fundamental in the modeling of complexity. Our results show that this distinction can be made regardless of the underlying system, providing a detailed description and insight on the dynamics, and most importantly without requiring multiple controlled experiments, as is common practice in previous applications of the KIM on neuron populations. This result opens to the adoption of the model in contexts where running repeated experiments is costly or impossible.

In conclusion, the Score-Driven KIM poses the foundations for a new modeling paradigm in complex systems. We foresee several relevant extensions such as the modeling of non binary data, for example extending to a Potts-like model^61^, or to non-Markovian settings. The key advantages provided by the score-driven methodology in terms of ease of estimation and minimization of model misspecification errors open to the implementation of more accurate and versatile models, in a wide range of disciplines that look to describe and unravel complexity from empirical observations.

## Methods

### Model inference

The KIM static parameters $\Theta$ are inferred via Maximum Likelihood Estimation using a known Mean Field technique^35^ or, when this is not possible, via standard Gradient Descent methods. Given $\Theta$ we estimate *w*, *B*, *A* by performing a targeted estimation^62^ through ADAM stochastic Gradient Descent^63^. Targeted estimation, which is common in observation-driven models such as the GARCH^64^, first fits the mean value of the time-varying parameter $\langle f \rangle = w/(1-B)$ and then fits the (*w*, *B*, *A*) parameters keeping this ratio constant. This procedure significantly reduces the estimation time and produces accurate estimates in our simulations. Further details on the process can be found in the SI.

A Lagrange Multiplier test^42^ is used to reject the hypothesis of constant parameters. The test statistic can be written as the Explained Sum of Squares of the auxiliary linear regression

9 $$\begin{aligned} \textbf{1} = c_w \nabla ^{0}_{t} + c_{A} S_{(t-1)}^0 \nabla _{t}^{0} \end{aligned}$$

where $\nabla ^{0}_{t}$ is the score at time *t* under the null hypothesis that $f(t) = w \; \forall \, t$, $S_{t}^0$ is the rescaled score (i.e. $\mathscr{I}^{-1/2}(t) \nabla _t$) at time *t* under the null, the constants $c_w$ and $c_A$ are estimated by standard linear regression methods and the resulting test statistic is distributed as a $\chi ^2$ random variable with one degree of freedom. If the null is rejected, the hypothesis that $\beta$ is time varying is a valid alternative and we can proceed to estimate the score-driven dynamics parameters. In the DyEnKIM, having multiple time-varying parameters, we test each parameter against two null hypotheses, one where all parameters are constant and one where all other parameters are score-driven, applying Benjamini-Hochberg^65^ correction for multiple tests. All tests on models presented here reject the null with $p < 0.001$.

### Codes

Computer codes for simulation and estimation of the models presented here are available at the persistent repository https://zenodo.org/badge/latestdoi/339464576.

### Data

US stock prices data provided by LOBSTER academic data - powered by NASDAQ OMX. The data consists of the reconstructed Limit Order Book for each US stock with timestamps at millisecond precision. We take the mid-price (*i.e.* the average between the best ask and the best bid prices in the Book) as a real-time proxy of the price, as done in Rambaldi et al.^40^. Time is discretized in 5 seconds time intervals to obtain a set of variables that have unconditional mean as close to 0 as possible, resulting in a balanced dataset.

The data describing situations of face to face proximity between individuals in the workplace, is provided by the SocioPatterns (http://www.sociopatterns.org/) collaboration. It was collected, over a period of two weeks, in one of the two office buildings of the Institut National de Veille Sanitaire, located in Saint Maurice near Paris, France. Two thirds of the total staff agreed to participate to the data collection. They were asked to wear a sensor on their chest, that allow exchange of radio packets only when the persons are facing each other at a range closer than 1.5 m. By design, any contact that lasted at least 20 seconds was recorded with a probability higher than 99%. In our temporal network application, we associate a node to each individual, and assign a link between two workers if they face each other at a distance less than 1.5 meters. We then consider only the subset of the 100 most active links in each day.

The salamander retina neuron data has been collected by Prof. Michael J. Berry II and made publicly available at *doi:10.15479/AT:ISTA:61*. It consists of measurements from 160 salamander retina ganglion cells collected through a multi-electrode array. The cells are responding to a light stimulus in the form of a 20 s naturalistic movie and the experiment is repeated 297 times. The electrical signal has been preprocessed to obtain a binary time series for each neuron with time resolution of 20 ms, identifying time intervals where the neuron has produced at least one spike with a 1, and -1 otherwise. From the public dataset we selected the 40 neurons with highest average spike rate over the 297 repeats.

## Supplementary Information

Supplementary Information.

## Data Availability

The financial data that support the findings of this study are available from LOBSTER Academic Data (https://lobsterdata.com) but restrictions apply to the availability of these data, which were used under license for the current study, and so are not publicly available. Data are however available from the authors upon reasonable request and with permission of LOBSTER Academic Data. The salamander retina neuron data analysed during the current study are available in the ISTA repository, https://doi.org/10.15479/AT:ISTA:61. The social contact network data analysed during the current study are available in the Sociopatterns repository http://www.sociopatterns.org/datasets/contacts-in-a-workplace/.
