# Supplementary material for: Modelling time-varying interactions in complex systems: the Score Driven Kinetic Ising Model
Source: Sci Rep. 2022 Nov 11;12:19339. doi: 10.1038/s41598-022-23770-0 (PMC9652375; doi:10.1038/s41598-022-23770-0)
Supplement: Supplementary file 1 — Supplementary Information. [file 41598_2022_23770_MOESM1_ESM.pdf]

Supplementary Information: Modelling time-varying interactions in complex systems: the Score-Driven Kinetic Ising Model

Carlo Campajola[1,2,3,*], Domenico Di Gangi[4], Fabrizio Lillo[1,5], and Daniele Tantari[5]

[1]Scuola Normale Superiore, p. dei Cavalieri 7, 56126 Pisa, Italy
[2]UZH Blockchain Center, University of Zürich, Rämistrasse 71, 8006 Zürich, Switzerland
[3]Department of Informatics, University of Zürich, Andreasstrasse 15, 8050 Zürich, Switzerland
[4]Institute of Information Science and Technologies, National Research Council (CNR), via G. Moruzzi 1, 56124 Pisa, Italy
[5]Department of Mathematics, University of Bologna, p. di Porta San Donato 5, 40126 Bologna, Italy

September 27, 2022

Additional information on Kinetic Ising Models

Spin systems have been analyzed by physicists since the early 20th century, mostly as models to understand the microscopical foundations of magnetism. A spin is indeed a proxy for an atomic magnetic moment, i.e. the torque the atom is subject to when immersed in a magnetic field. The first spin model is the celebrated 1D Ising Model [1], which mathematically abstracts the problem to an infinite chain of binary variables s (the spins) that can be either in an "up" or "down" state. These perceive the local magnetic field generated by their nearest neighbors as well as any external magnetic field and tend to "align" (i.e. match the state) or "disalign" (i.e. go in the opposite state) with the net field they sense, based on the value of a parameter J. The model was intended to verify the hypothesis that thermal properties of ferromagnetic materials can arise from microscopic interactions between their atoms, but failed to do so because no net magnetic field would be observed at equilibrium. However, it was later shown [2] that the failure was not due to the mechanism, but to the oversimplification of taking a 1-dimensional system: in fact, if one takes a 2D lattice instead of a 1D chain, this extremely simple model qualitatively reproduces the macroscopic thermal properties of ferromagnets. The success of the Ising Model has led to its extension and refinement to describe exotic materials such as spin glasses [3], and its fascinating ability to describe macroscopic properties determined by microscopic coordination posed the foundations to many quantitative models of

complex systems, with examples of successful Ising-like models for protein and DNA chains [4], neurons [5] and financial markets [6].

The appeal of Ising Models comes in part from the fact that they belong to the class of *Maximum Entropy* models, as introduced by [7]. The principle states that, given a set of constrained quantities from available observations - such as sample averages - a probability distribution that maximizes Shannon's entropy [8] subject to the constraints is the best distribution to describe the observations, as it is the one that makes the least arbitrary assumptions. In particular Ising Models result from Shannon's entropy maximization constraining means and correlations of the spins, thus making them a popular choice to describe systems that can be encoded in binary strings.

The Kinetic Ising Model (KIM) is the out-of-equilibrium version of the Sherrington-Kirkpatrick (SK) spin glass [9, 10], developed a few years later and proposed as dynamical model for asymmetric neural networks with discrete time and synchronous sampling. The model's transition probability, describing the probability of observing a future configuration $\{s_i(t)\}$ given a current configuration $\{s_t(t-1)\}$, reads

$$p\left(\{s_i(t)\}|\{s_i(t-1)\}, J, h\right) = \frac{1}{Z(t)} \exp\left\{\sum_{i,j} J_{ij} s_i(t) s_j(t-1) + \sum_i h_i s_i(t)\right\} \tag{1}$$

Differently from its predecessor, which describes the equilibrium properties of spin glasses, this model describes the dynamics of a system of spins which have asymmetric interactions, namely spin i's effect on spin j is different from spin j's effect on i. This difference is incorporated in the structure of the J matrix, which is symmetric in the SK model and asymmetric in the KIM. Having $J_{ij} \neq J_{ji}$ in fact implies that these coefficients can no longer describe a synchronous interaction, as for instance a correlation coefficient, but need to describe an asynchronous one, specifically in this case a lag one interaction.

Typically, in the physics literature, the J elements are assumed to be *iid* Gaussian random variables, $J_{ij} \sim \mathcal{N}(\mu_J/N, \sigma_J^2/N)$ and the properties of the model as data generating process are the object of analysis. As shown in [10], the KIM loses the so-called "spin glass" phase of the SK model - a phase in which the system "freezes" in a metastable configuration with local order but no global order - and only presents a dynamic phase transition between a paramagnetic phase - where spins do not show preferential alignment - and a ferromagnetic phase - where all spins align in one direction - when the mean of the J elements, μ_J/N, is greater than $1/N$.

A more complete characterization of the model can be found in the physics literature [10, 9, 11, 12], with recent developments contributing to neuroscience [13], machine learning [14, 15, 16] and finance [17] literatures. As a last remark, the model has been developed in at least another independent strand of literature with the name of Discrete AutoRegressive model (DAR) [18], and an equivalence between these models has been recently shown in [19].

Additional information on score-driven models

Let us set the stage to better explain score-driven models by briefly reviewing the theory of time-varying parameters models in discrete time. There is a rich literature on the topic, which has been summarized in the review by Tucci [20] and more recently by Koopman et al. [21]. In general, a time-varying parameters model can be written as

$$s(t) \sim p(s(t)|f(t), S(t-1), \Phi_1) \tag{2a}$$

$$f(t) = \psi(f(t-1), f(t-2), ..., S(t-1), \epsilon(t), \Phi_2) \tag{2b}$$

where $s(t)$ is a vector of observations sampled from the probability distribution function p, $S(t-1)$ is the set of all observations up to time $t-1$ and $f(t)$ are the parameters which are assumed to be time varying. The dynamics of those parameters can either depend on past observations, on past values of the same parameters, on some external noise $\epsilon(t)$ and on two sets of static parameters Φ_1 and Φ_2.

If the function ψ only contains past values of the time-varying parameters, a noise term and the static parameters, then the model is called a *parameter-driven* model, whereas if the function ψ can be written as a deterministic function only of past observations and past parameters, it is called an *observation-driven* model [22].

Examples for parameter-driven models can be found in the financial econometrics literature looking at stochastic volatility models [23, 24], which aim at describing the time-varying nature of the volatility (*i.e.* the variance) of price variations, as well as other examples [25, 26].

Within the observation-driven models, the most celebrated example is the Generalized AutoRegressive Conditional Heteroscedasticity (GARCH) model [27], where a time series of financial log-returns is modelled using a time-varying volatility parameter depending deterministically on squared observations up to that time and past values of volatilities.

The main advantage of adopting an observation-driven model rather than a parameter-driven one lies in its estimation: having time-varying parameters that only depend on observations through a set of static parameters results in a strong reduction of complexity in writing the likelihood of the model, whereas the calculations for most non-trivial parameter-driven models are typically extremely convoluted and computationally intensive.

Score-driven (or Generalized Autoregressive Score - GAS - models) are a specific class of observation-driven models. Originally introduced by Creal et al. [28] and Harvey [29], they postulate that time-varying parameters depend on observations through the score of the conditional likelihood, that the gradient of its logarithm.

Let us restate Eq. 2 of the main text to provide a more detailed explanation of the score-driven dynamics

$$f(t+1) = w + Bf(t) + A\mathcal{I}^{-1/2}(t)\nabla_t \tag{3}$$

where w, B and A are a set of static parameters, $f(t) \in \mathbb{R}^M$ is a vector of time-varying parameters of the model's conditional probability density $p(s(t)|f(t), ...)$ with $s(t) \in \mathbb{R}^N$, and $\nabla_t = \frac{\partial \log p(s(t)|f(t))}{\partial f(t)}$ is the score. In this generic form, w

is a M-dimensional vector, while A and B are $M \times M$ matrices. $\mathcal{I}^{-1/2}(t)$ is also a $M \times M$ matrix, introduced to rescale the time t score to account for local convexity, that we choose to be the inverse of the square root of the Fisher information matrix associated with $p(s(t)|f(t))$. This is not the only possible choice for this rescaling matrix [28] but in our opinion it is the most intuitive way of rescaling the score (and probably the most common one).

As mentioned, one of the main reasons to choose an observation-driven model is the less challenging estimation, but it is not the only one. Score-driven models in particular have been shown to be optimal in terms of Kullback-Leibler divergence [30, 31] in approximating any unknown underlying probability distribution. Given the absence of unobservable noise processes, contrary to parameter-driven models in general, they are able to properly fit unknown parameter dynamics with accuracy that is second only to the data generating process itself. This makes score-driven models the ideal choice whenever prior knowledge about parameters dynamics is scarce.

Finally, the score-driven modelling approach provides access to a simple Lagrange Multiplier statistical test [32], of the null hypothesis that a given parameter is constant. This is of crucial importance when estimating a model parameters from data, as knowing whether the parameter can be considered static or should be assumed to be time-varying helps in the selection of models that extract more relevant informations from the data and are less prone to overfitting or underfitting problems. We reported details on the Lagrange Multiplier test in the "Methods" section in the main text.

Score-Driven models as filters of a misspecified dynamics

Here we present a simple example of how score-driven models can be used to filter an unknown dynamics of a parameter, without assuming a specific model for its time evolution. We will do it by considering the classical case of a discrete time random walk model with time-varying diffusion coefficient. This type of models is very popular in finance where the (logarithm of the) price follows a random walk and the diffusion rate, termed volatility, represents the risk of the asset. As we will show, under minimal assumptions, such a filter turns out to coincide with the popular GARCH model for volatility.

All this is relatively well known. Indeed, the interpretation of GARCH processes as predictive filters is well described in this statement by Nelson [33]: "Note that our use of the term 'estimate' corresponds to its use in the filtering literature rather than the statistics literature; that is, an ARCH model with (given) fixed parameters produces 'estimates' of the true underlying conditional covariance matrix at each point in time in the same sense that a Kalman filter produces 'estimates' of unobserved state variables in a linear system".

Let us call $s(t)$ the increment of the log-price, $p(t+1) - p(t)$ and consider a stochastic volatility model

$$s(t) = \sigma(t)\epsilon(t) \qquad \epsilon(t) \sim \mathcal{N}(0,1).$$

i.e. the conditional probability density function of $s(t)$ is

$$p(s(t)|\sigma(t)) = \frac{1}{\sqrt{2\pi\sigma^2(t)}} e^{-\frac{s^2(t)}{2\sigma^2(t)}}$$

By choosing as time-varying parameter $f(t) = \sigma^2(t)$, the score of the likelihood is

$$\frac{\partial \log p(s(t)|f(t))}{\partial f(t)} = -\frac{1}{2\sigma^2(t)} + \frac{s^2(t)}{2\sigma^4(t)}$$

hence the equation for the evolution of volatility is

$$\sigma^2(t+1) = w + B\sigma^2(t) + \frac{A\mathcal{I}^{-1/2}(t)}{2}\left[\frac{s^2(t) - \sigma^2(t)}{\sigma^4(t)}\right]$$

Thus if $s^2(t) \gg \sigma^2(t)$ ($s^2(t) \ll \sigma^2(t)$), the new $\sigma^2(t+1)$ will be larger (smaller) than $\sigma^2(t)$. This is exactly the mechanism which dynamically adjusts the filtered estimation of volatility taking into account the most recent observation(s).

By choosing $\mathcal{I}(t)$ as the Fisher information matrix and using $\mathbb{E}[s^2(t)|\sigma^2(t)] = \sigma^2(t)$, we find

$$\mathcal{I}(t) \equiv -\mathbb{E}\left[\frac{\partial^2 \log p(s(t)|\sigma(t))}{\partial^2\sigma^2(t)}\bigg|\sigma^2(t)\right] = -\mathbb{E}\left[\frac{1}{2\sigma^4(t)} - \frac{s^2(t)}{\sigma^6(t)}\bigg|\sigma^2(t)\right] = \frac{1}{2\sigma^4(t)}$$

and thus

$$\sigma^2(t+1) = w + B\sigma^2(t) + A(s^2(t) - \sigma^2(t)) = w + \alpha s^2(t) + \beta\sigma^2(t) \qquad (4)$$

with $\alpha = A$ and $\beta = B - A$, which coincides with the GARCH model. This model has been originally proposed as a data generating process for describing realistic dynamics of volatility, while here it is derived as a result of Score-Driven modeling. The GARCH model of Eq. 4 is typically seen as a data generating process for the volatility, and thus the price, of financial assets. This model is routinely estimated from real data and used widely in the financial industry for risk management, portfolio allocation, systemic risk, etc.. Fig. S1 shows a typical simulated price pattern from a GARCH(1,1) process, displaying fat tails and clustered volatility, as observed in empirical data. Other popular econometric models as Multiplicative Error Model (MEM), Autoregressive Conditional Duration (ACD), Autoregressive Conditional Intensity (ACI) can be cast as special cases of score-driven models.

However, the main point we want to make here concerns the use of GARCH, and more generally of score-driven models, as *filters* of a differently specified dynamics. To show this in practice, we simulate 1000 price observations from the model

$$s(t) = \sigma(t)\epsilon(t) \qquad \epsilon(t) \sim \mathcal{N}(0,1) \qquad \sigma(t) = 2 + \frac{1}{2}\sin\left(\frac{\pi t}{100}\right) \qquad (5)$$

The left panel of Fig. S2 shows the simulated price dynamics. This is clearly *not* a GARCH model and the sinuisodal shape can be modified with other deterministic or stochastic processes. Assuming the data generating process of Eq. 5 is unknown, one can nevertheless fit the GARCH(1,1) model and obtain, beside the static parameters w, α, and β, the filtered values of $\sigma(t)$. The outcome of this procedure is shown in the right panel of Fig. S2 where the red line is the simulated $\sigma(t)$, while the black circles represent the filtered values of $\sigma(t)$.

The example shows how score-driven models can be used to filter the time-varying parameters with unknown dynamics from data. As mentioned in the main text, Score-driven models have been shown to be an optimal choice among observation-driven models when minimising the Kullback-Leibler divergence to an unknown generating probability distribution [30].

Details on the inference method

As mentioned in the main text our estimation procedure is done in steps, starting by estimating the parameters $\Theta = (J, h)$ of the standard KIM and then running a targeted estimation for the w, B and A parameters. In this Appendix we provide some further details about this procedure.

The whole process can be summarized as the maximization of the log-likelihood $\mathcal{L}(\Theta, \beta(t), w, B, A)$ of the model in question, which in the case of the DyNoKIM reads (setting as usual $h_i = 0 \ \forall i$)

$$\mathcal{L}(\Theta, \beta(t), w, B, A) = \sum_{t=1}^{T} \left\{ \sum_i \left[\beta(t) \sum_j s_i(t) J_{ij} s_j(t-1) \right] - \log Z(t) \right\} \quad (6a)$$

$$\text{with} \quad \log \beta(t+1) = w + B \log \beta(t) + A \mathcal{I}^{-1/2}(t) \nabla_t \quad (6b)$$

and the definitions of the various quantities are given in the main text. The log-likelihood shown above has a recursive form, as each term in the sum of Eq. 6a depends on $\beta(t)$, which is determined recursively through Eq. 6b from a starting condition $\beta(1)$. This means that, if one were to maximize $\mathcal{L}$ with respect to all the parameters by applying a standard Gradient Descent method, at each computation of $\mathcal{L}$ and its gradient it would be necessary to compute the recursion, resulting in a slow and computationally cumbersome process. In order to make the estimation quicker we implement our multi-step procedure, relying on existing methods for the estimation of the standard KIM and of observation-driven models.

Our first step consists of maximizing $\mathcal{L}$ with respect to the standard KIM parameters Θ. This is done adopting the Mean Field approach of Mézard and Sakellariou [34], which is both fast and accurate in the estimation of fully connected models. We refer the interested readers to the original publication for further details on the method itself. The main reason to detach this step from the optimization of the complete log-likelihood is that Θ contains a large number of parameters: if one can get an estimate for those without recurring to slow and hard to tune Gradient Descent methods the computational cost of the inference reduces significantly.

Given the values of Θ obtained in the first step, we then move to the targeted estimation of w, B and A. This consists in first estimating a target value $\bar{f}$ for the unconditional mean of $f(t) = \log \beta(t)$ and then optimize w, B and A maintaining the ratio $w/(1-B) = \bar{f}$ fixed. To estimate $\bar{f}$ we maximize the log-likelihood of Eq. 6a temporarily imposing $A = B = 0$, hence Eq. 6b becomes $\log \beta(t) = \bar{f} = \text{const}$. Finally, given this target value we optimize $\mathcal{L}$ with respect to w, B and A maintaining the ratio $w/(1-B) = \bar{f}$ fixed and setting $f(1) = \bar{f}$ to start the recursion of Eq. 6b. During these last two steps we use the ADAptive Momentum (ADAM) [35] Stochastic Gradient Descent method as optimization algorithm, as we found in our case it had better performance with respect to other available methods.

This targeted estimation is not necessary - one could directly estimate w, B and A together - but it is a standard procedure in the estimation of observation-driven models like the GARCH [36], as it typically reduces the total number of iterations of gradient descent.

We point out one last remark concerning the indetermination of $\langle\beta\rangle$ in the DyNoKIM of Eq. 6 in the main text, which is crucial to understand the results of our simulations. The fact that these values cannot be identified is not problematic *per se*, but requires caution when comparing models and filtered parameters across different samples, or when comparing estimates with simulations. To avoid misleading results, one needs to enforce the sample mean of the filtered $\beta(t)$ (or of each of the elements of $\boldsymbol{\beta}(t)$ in the DyEnKIM) to be equal to a reference value, which without loss of generality we pick to be $\langle\beta\rangle = 1$. This is easily done by running the estimation and filtering, then measuring $\langle\beta\rangle$ and rescaling $\beta'(t) = \beta(t)/\langle\beta\rangle$. To leave the model unchanged an opposite rescaling is needed for the parameters J and h, each having to be multiplied by $\langle\beta\rangle$ themselves. This transformation does not change the log-likelihood, thus the model parameters are still MLE, but crucially allows to set a reference value for β that solves the indetermination.

Given this remark, in all the simulations we show where the data generating process of $\beta(t)$ is misspecified we generate its values making sure that their sample mean is 1. By doing so we do not lose any generality in our results, as the indetermination needs to be solved for the data generating process too if one wants to obtain meaningful results, and we are able to correctly compare the simulated values of J, h and $\beta(t)$ with the ones that are estimated by the score-driven model. Notably, since the model is misspecified, this cannot be achieved during estimation by enforcing the targeted unconditional mean to be equal to 1, as the score in that case is not a martingale difference and thus the unconditional mean of the score-driven parameter is ill-defined itself, as shown by Creal et al. [28].

Derivation of the theoretical AUC

Here we expand on the derivation of the theoretical Area Under the ROC Curve shown in Fig. 1 in the main text. A ROC curve is a set of points $(FPR(\alpha), TPR(\alpha))$, with $\alpha \in [0, 1]$ being a free parameter determining the minimum value of $p(s_i(t) = +1|s(t-1); \beta, \Theta)$ which is considered to predict $\hat{s}_i(t) = 1$. If the prediction $\hat{s}_i(t)$ matches the realization $s_i(t)$ then the classification is identified as a True Positive (or Negative, if $p < \alpha$), otherwise it is identified as a False Positive (Negative). The True Positive Rate (TPR) is the ratio of True Positives to the total number of realized Positives, that is True Positives plus False Negatives. Similarly the False Positive Rate (FPR) is the ratio of False Positives to the total number of realized Negatives. Summarizing

$$TPR = \frac{TP}{TP + FN}; \qquad FPR = \frac{FP}{FP + TN}$$

We can explicitly derive the analytical form of the theoretical Area Under the Curve, that is the area that lies below the set of points $(FPR(\alpha), TPR(\alpha))$, assuming the data generating process is well specified and performing some assumptions on the distribution of the model parameters. As a reminder, a classifier having $AUC = 0.5$ is called an *uninformed classifier*, meaning it makes predictions statistically indistinguishable from random guessing, while values of AUC greater than 0.5 are a sign of good forecasting capability.

Following the definition of TPR and FPR one can compute their expected values

$$TPR_\phi(\alpha,\beta) = \frac{1}{Z^+_\phi(\beta)} \int_{g_i:p^+>\alpha} dg_i \phi(g) p^+(\beta, g_i) \tag{7a}$$

$$FPR_\phi(\alpha,\beta) = \frac{1}{Z^-_\phi(\beta)} \int_{g_i:p^+>\alpha} dg_i \phi(g) p^-(\beta, g_i) \tag{7b}$$

where $Z^\pm_\phi(\beta) = p(s_i = \pm 1)$ is a normalization function, $\phi(g)$ is the unconditional distribution of the effective fields g_i and we have abbreviated the probability of sampling a positive or negative value as

$$p^\pm(\beta, g_i) = \frac{e^{\pm\beta g_i}}{2\cosh(\beta g_i)}$$

The definition of the theoretical AUC then reads as

$$AUC_\phi(\beta) = \int_1^0 TPR_\phi(\alpha,\beta) \frac{\partial FPR_\phi(\alpha,\beta)}{\partial \alpha} d\alpha$$

that is the area below the set of points $(FPR(\alpha), TPR(\alpha))$. The lower limit to the integration in Eqs. 7 is $g_{min} : p^+(g_{min}) = \alpha$, which is found to be

$$g_{min}(\alpha,\beta) = \frac{1}{2\beta} \log \frac{\alpha}{1-\alpha}$$

Then applying the partial derivative to the definition of FPR it follows that

$$\frac{\partial FPR}{\partial \alpha} = -\frac{1}{Z^-_\phi(\beta)} \frac{\partial g_{min}}{\partial \alpha} \phi(g_{min})(1-\alpha)$$

where we have substituted $p^-(\beta, g_{min}) = 1 - \alpha$. Plugging all the above results in the definition of AUC_ϕ we then find

$$AUC_\phi(\beta) = \frac{1}{Z^+_\phi(\beta) Z^-_\phi(\beta)} \int_0^1 d\alpha \left[\int_{g_{min}(\alpha,\beta)}^{+\infty} dg \phi(g) \frac{e^{\beta g}}{2\cosh \beta g} \right] \left[\frac{1}{2\alpha\beta} \phi(g_{min}(\alpha,\beta)) \right] \tag{8}$$

From an operational perspective $\phi(g)$ is the distribution that the effective fields show cross-sectionally across the whole sample, that is $g_i(t) \sim \phi(g)\ \forall i, t$, but it can also be calculated by giving a prior distribution to the static parameters of the model, $\Theta = (J, h, b)$. Finding this distribution can be useful to provide an easier and more accurate evaluation of the expected AUC of a forecast at a given β value, as it provides a bridge from the model parameters to the $AUC(\beta)$ we derived in Eq. 8 and shown in Fig. 1 in the main text.

Let us assume, as is standard in the literature [10, 37, 34], that the parameters Θ are structured in such a way that

$$J_{ij} \overset{iid}{\sim} \mathcal{N}(\mu_J/N, \sigma_J^2/N - \mu_J^2/N^2)$$
$$h_i \overset{iid}{\sim} \mathcal{N}(\mu_h, \sigma_h^2)$$

If that is the case then the distribution of $g_i(t)$ is itself a Gaussian, as $g_i(t)$ is now a sum of independent Gaussian random variables J_{ij} and h_i with random coefficients $s_j(t)$. Let us also define two average operators: the average $\langle\cdot\rangle$ over the distribution p, also called the *thermal* average (which, the system being ergodic, coincides with a time average for $T \to \infty$), and the average $\overline{\cdot}$ over the distribution of parameters, also known as the *disorder* average. Following Mézard and Sakellariou [34] we can then find the unconditional mean of s_i which reads

$$m_i = \langle s_i(t)\rangle = \langle \tanh\left[\beta g_i(t)\right]\rangle \tag{9}$$

where we have substituted the conditional mean value of $s_i(t)$ inside the brackets. This depends from the distribution of $g_i(t)$: assuming stationarity and calling $g_i^0 = \langle g_i(t)\rangle$ and $\Delta_i^2 = \langle g_i^2(t)\rangle - \langle g_i(t)\rangle^2$ we find that they are

$$g_i^0 = \langle \sum_j J_{ij} s_j(t) + h_i \rangle = \sum_j J_{ij} m_j + h_i \tag{10a}$$

$$\Delta_i^2 = \left\langle \left(\sum_j J_{ij} s_j(t) + h_i \right)^2 \right\rangle - \left\langle \sum_j J_{ij} s_j(t) + h_i \right\rangle^2 = \sum_{j,k} J_{ij} J_{ik} \left[\langle s_j(t) s_k(t)\rangle - m_j m_k\right] \tag{10b}$$

In Eq. 10b spins $s_j(t)$ and $s_k(t)$ are mutually conditionally independent under distribution p: this means that the only surviving terms are the ones for $j = k$, and thus we find

$$\Delta_i^2 = \sum_j J_{ij}^2 (1 - m_j^2) \tag{11}$$

Having determined the value of the mean and variance of the effective field of spin i we can now proceed to average over the disorder and find the unconditional distribution of effective fields at any time and for any spin, $\phi(g)$. First we realize that the average of Eq. 9 can now be substituted by a Gaussian integral

$$m_i = \int Dx \tanh\left[\beta\left(g_i^0 + x\Delta_i\right)\right] \tag{12}$$

where Dx is a Gaussian measure of variable $x \sim \mathcal{N}(0,1)$. Then we can see that the unconditional mean of the fields distribution $\phi(g)$ is

$$\mu_g = \overline{\langle g_i(t)\rangle} = \overline{\sum_j J_{ij} m_j + h_i} \tag{13}$$

Given the above results and the definition of J, the dependency between J_{ij} and m_j vanishes like $O(1/N)$, which means that the two can be averaged over the disorder separately in the limit $N \to \infty$. This results in the following expression for the unconditional mean of $g_i(t)$

$$\mu_g = \mu_J \overline{m_j} + \mu_h = \mu_J m + \mu_h \tag{14}$$

where

$$m = \overline{m_i} = \overline{\int Dx \tanh\left[\beta(g_i + x\Delta_i)\right]}$$

both the integral and the average here are of difficult solution and results have been provided by Crisanti and Sompolinsky [10]: they show that in the limit $N \to \infty$ and with $h_i = 0\ \forall i$ the system can be in one of two phases, a paramagnetic phase where $m = 0$ if β is smaller than a critical threshold $\beta_c(\mu_J)$ and $\mu_J < 1$, and a ferromagnetic phase where $m \neq 0$ otherwise. In the following we report results for simulations in the paramagnetic phase, as the inference is not possible in the ferromagnetic phase. To give better intuition let us consider the integral above in the limit $\beta \to 0$: then we can expand the hyperbolic tangent around 0 to find (since x has zero mean)

$$m \approx \overline{\beta g_i} = \overline{\beta \left(\sum_j J_{ij} m_j + \mu_h \right)} = \beta(\mu_J m + \mu_h) \tag{15}$$

which in turn leads to an approximated solution for μ_g in the limit $\beta \to 0$

$$\mu_g \approx \mu_h \left(\frac{\beta \mu_J}{1 - \beta \mu_J} + 1 \right)$$

Moving on to the variance of g the calculation is straightforward. Adding the mean over the disorder to Eq. 10b we find

$$\begin{aligned}
\sigma_g^2 &= \overline{\left\langle \left[\sum_j J_{ij} s_j(t) + h_i \right]^2 \right\rangle} - \overline{\left\langle \sum_j J_{ij} s_j(t) + h_i \right\rangle}^2 = \\
&= \overline{\sum_j J_{ij}^2 + h_i^2 + 2h_i \sum_j J_{ij} m_j} - \overline{\sum_j J_{ij} m_j + h_i}^2 = \\
&= \sigma_J^2 + \sigma_h^2 - \mu_J^2 m^2
\end{aligned} \tag{16}$$

Equations 14 and 16 can then be used to calculate, given the parameters of the distribution generating Θ, the values of μ_g and σ_g that are to be plugged in the distribution $\phi(g)$ of Eq. 8

We simulated a Kinetic Ising Model with $N = 100$ spins for $T = 2000$ time steps at different constant values of β and then measured the AUC of predictions assuming the parameters are known. In Fig. S3 we report a comparison between these simulated values and the theoretical ones provided by Eq. 8 varying β and the hyperparameters μ_J, σ_J, μ_h and σ_h in the Gaussian setting we just discussed and adopting the expansion for $\beta \to 0$. We see that the approximation for small β of Eq. 15 does not affect the accuracy of the theoretical prediction for larger values of β and that the mean is correctly captured by Eq. 8. The only exception to this is found for $\beta > 1$ and $\mu_J = 1$, which according to the literature is close to the line of the ferromagnetic transition: in this case the small β approximation fails to predict the simulated values. Larger values of N and T (not shown here) produce narrower error bars.

The general effect we see from Fig. S3 is that higher variance of the J and h parameters leads to higher AUC values leaving all else unchanged (orange squares and yellow circles), while moving the means has little effect as long as the system is in its paramagnetic phase.

These results are easy to obtain thanks to the assumption that the model parameters J and h have Gaussian distributed entries, but in principle the

distribution $\phi(g)$ can be derived also for other distributions, albeit probably requiring numerical solutions rather than the analytical ones we presented here.

Further details on the DyEnKIM

There are a couple of subtleties that need to be pointed out regarding the structure of the B and A parameters and of the Fisher Information $\mathcal{I}$ of the DyEnKIM, which are matrices rather than scalars as in the case of the DyNoKIM.

In order to make the estimation less computationally demanding in our example applications we assume A, B and $\mathcal{I}$ diagonal, disregarding the dependencies between time-varying parameters: this will likely make our estimates less precise, but it also reduces the number of static parameters to be inferred, letting us bypass model selection decisions which are outside the scope of this article.

As previously discussed there is also in this case the problem of identification for the averages of the components of $\boldsymbol{\beta} = (\beta_{diag}, \beta_{off}, \beta_h)$, which we solve in the exact same way as we did for the DyNoKIM by dividing the values of each component by their sample mean while multiplying the associated static parameter by the same factor, again leaving the likelihood of the model unchanged, but setting a reference level for $\boldsymbol{\beta}$.

As a last remark, notice that the DyNoKIM and the DyEnKIM are equivalent when $h_0(t) = 0 \;\forall\, t$ and $\beta_{diag} = \beta_{off} = \beta_h = \beta$. For this reason we mainly present simulation results for the DyNoKIM alone to keep the manuscript concise, as we found no significant differences between the two models when it comes to the reliability of the estimation process.

Consistency analysis for estimation

We perform a consistency test on simulated data, aimed at understanding whether the two-step estimation procedure we outlined above is able to recover the values of the parameters of the model when the model itself generated the data.

Here we report results for simulations run with parameters $N = 50$, $T = 750$ or $T = 1500$, $J_{ij} \sim \mathcal{N}(0, 1/\sqrt{N})$, $h_i = 0 \;\forall\, i$, $B = 0.95$ and $A = 0.01$. We see from Fig. S4 that the estimation of the elements of J is indeed consistent: we estimate a linear regression model between the estimated and the true values of J_{ij}, namely $J_{ij}^{est} = a + bJ_{ij}^{true} + \epsilon$, and plot the histogram of the values of b and of the coefficient of determination R^2 of the resulting model from 250 simulations and estimations (a is consistently found to be very close to 0 in all our simulations and for this reason we omit it). In the ideal case where for any i, j $J_{ij}^{est} = J_{ij}^{true}$ one would have $b = R^2 = 1$, which is what we aim for in the limit $T \to \infty$. We see from our results that there is indeed a convergence of both values towards 1 when increasing sample size, reducing both the bias and the variance of the regression parameters.

Turning to the score-driven dynamics parameters A and B, the situation does not change significantly. In Fig. S5 we show the histograms of estimated values of B and A over 250 simulations of $N = 50$ variables for both $T = 750$ and $T = 1500$. It again appears clearly that when increasing the sample size the bias and variance of the estimators converge towards 0, with the estimated parameter converging towards its simulated value. Thanks to these results we

are able to confidently apply the two-step estimation method without the need to estimate all the parameters at once.

To add further evidence to what we presented in the main text, here we also report two additional figures regarding the filtering of misspecified $\beta(t)$ for the DyNoKIM and the DyEnKIM. In Fig. S6 we show two examples of misspecified $\beta(t)$ dynamics that are correctly recovered by the score-driven approach: the first is a deterministic sine wave function and the second is an AutoRegressive model of order 1 (AR(1)) which follows the equation

$$\beta^{AR}(t+1) = a_0 + a_1 \beta^{AR}(t) + \epsilon(t)$$

where $\epsilon(t) \sim \mathcal{N}(0, \Sigma^2)$ with parameters $a_0 = 0.005$, $a_1 = 0.995$, $\Sigma = 0.01$ so to have $\langle \beta^{AR} \rangle = 1$ and we select a simulation where $\beta(t) > 0 \; \forall \; t$. In both cases we simulate 30 time series of length T using the given values of $\beta(t)$ to generate the $s(t)$; given only the simulated $s(t)$ time series, the inference algorithm determines the optimal static parameters A, B and J and filters the optimal value of $\beta(t)$ at each time. We see that regardless of whether the underlying true dynamics is deterministic, stochastic, or more or less smooth the filter is rather accurate in retrieving the simulated values.

Regarding the DyEnKIM we want to show that different effects are correctly separated and identified when estimating the model on a misspecified data generating process. In fact while the consistency analysis largely resembles the one we reported for the DyNoKIM in Figures S4 and S5 and for this reason we omit it, the effect of filtering multiple time-varying parameters is something that cannot be predicted by the simulations on the DyNoKIM alone.

In Figure S7 we show the results when estimating the DyEnKIM on a dataset generated by a Kinetic Ising Model with time-varying $\beta_{diag}(t)$, $\beta_{off}(t)$ and $\beta_h(t)$ but where the dynamics of the parameters is predetermined instead of following the score-driven update rule. We arbitrarily choose to take a constant $\beta_{diag}(t) = 1$, a piecewise constant $\beta_{off}(t)$ and an exponentiated sinusoidal $\beta_h(t) = \exp[\sin(\omega t)]$, with $\omega = 5\frac{2\pi}{T}$, $T = 1500$ and $N = 30$. The results show that the filter works correctly and that the different time-varying parameters are consistently estimated, regardless of the kind of dynamics given to each of them.

Additional results on neural population data

In this section we provide further results on the application of the KIM to the neural population data. In particular we discuss the possibility to use the DyEnKIM to test the significance of the elements of J fitted over multiple experiments and expand on the comparison between the static parameters KIM and the score-driven version.

In Figure S8a we show an example of the analyzed data in the form of a raster plot of one of the experiments. It appears clear that the dynamics of spikes is bursty and there are non-negligible auto- and cross-correlation effects among neurons, likely driven by the external stimulus of the video [38].

As mentioned in the section on the DyNoKIM in the main text, it is possible to use the filtered time-varying parameters to correct the values of J from misspecification error. The same can be done with the DyEnKIM, correcting the elements of J by a factor given by the sample mean of β_{diag} and β_{off} and the

external fields h by the sample mean of β_h. In Figure S8b we show a scatterplot of the values of J_{ij}, comparing the parameters fitted with a KIM on all available data (x-axis) with the average value $\overline{J}_{ij} = \frac{1}{M}\sum_k^M J_{ij}^{(k)}$, where $J_{ij}^{(k)}$ is the value fitted with the DyEnKIM correction on experiment k. It is clear that, as in the case of DyNoKIM shown in Figure 2 of the main text, the correction - which in this case only affects off-diagonal elements as $\beta_{diag} = 1$ - tends to increase the absolute value of J_{ij} with respect to the static KIM version.

Pruning irrelevant parameters is central to the definition of meaningful statistical models. Here we propose two alternative methods, Decimation and t-testing; the first is standard in the literature on Kinetic Ising Models [15], whereas the second exploits the repeated experiments in the data to compare parameters fitted on different samples and assess their significance by means of a t-test. For Decimation we refer the interested readers to the original paper introducing it [15]. As an alternative in cases where multiple repetitions of the experiment are available, as is the case for our neuron spike dataset, it is possible to fit a DyEnKIM for each of the M experiments and then use a Student's t-test on the set of values J_{ij}^k, $k = 1, \dots, M$ to test whether their average is significantly different from 0. In Figure S8c we show a comparison between these two methods, with Decimation applied to the KIM J and the t-test used to validate the DyEnKIM result, adopting a Bonferroni correction for multiple hypothesis testing at the $p < 0.01$ level. In our case, the Decimation approach selects less elements of J_{ij} as significant, whereas the t-test appears to be less specific or more sensitive. This difference is possibly related to Decimation being a likelihood-based method, which may suffer from misspecification in case the data generating process is not a KIM, but answering this question goes beyond the scope of this paper. Finally, in Figure S8d we report a visualization of the t-tested DyEnKIM $\overline{J}$ matrix. The diagonal elements are largely positive, indicating significant autocorrelation in spiking dynamics (as would be expected by a visual inspection of the raster plot), whereas off-diagonal elements are generally smaller in absolute value and both positive and negative, albeit significantly different from 0.

Details on the Relation Between KIM and TERGM

Here we discuss the relation between the Kinetic Ising Model (KIM) and the Temporal Exponential Random Graph (TERGM) of [39], and show how our score driven extensions can be seen as time varying parameters extensions of TERGM.

Considering only lag 1 dependencies, a TERGM is defined by the following probability mass function

$$P(G(t)|G(t-1), \theta) = \frac{e^{\sum \theta_l q_l(G(t), G(t-1))}}{K(\theta)}, \tag{17}$$

where the functions $q_l(G(t), G(t-1))$, called network statistics, are defined to investigate the determinants of the network's dynamics, and $K(\theta)$ is a normalization coefficient, known as partition function. Examples of network's statistics are $q_{stab}(G(t), G(t-1)) = \sum_{ij} G_{ij}(t)G_{ij}(t) + (1 - G_{ij}(t))(1 - G_{ij}(t-1))$, that captures links' stability, and $q_{dens}(G(t)) = \sum_{ij} G_{ij}(t)$, related to network's density.

As mentioned in the main text, we can easily map each entry of the adjacency matrix into a spin and associate a sequence of spins to a discrete time temporal network. Indicating by $vec(G)$ the vectorized version of matrix G, the mapping can be summarized by the relation

$$vec(G(t))_k = 1/2 + s_k(t)/2$$

and the KIM is equivalent to the following version of the TERGM

$$\begin{aligned} P(s(t)|s(t-1),\theta) &= \frac{e^{\sum_{ij} J_{ij} s_i(t-1) s_j(t) + \sum_i h_i s_i(t)}}{Z(J,h)} = \\ &= \frac{e^{\sum_{ab} \theta^{(1)}_{ab} vec(G(t-1))_a vec(G(t))_b + \sum_a \theta^{(2)}_a vec(G(t))_a - \sum_a \theta^{(3)}_a vec(G(t-1))_a}}{K(\theta)}, \end{aligned}$$

where we omitted the constant terms, not depending on the adjacency matrix, that have been absorbed in the normalization constant. Hence, KIM is equivalent to a TERGM having three kinds of network statistics: first, the set of all possible lagged interactions $q^{(1)}_{ab} = vec(G(t-1))_a vec(G(t))_b$ between pairs of links, each appearing in this specification of Eq. 17 with a parameter $\theta^{(1)}_{ab} = 4J_{ab}$; second, a term associated to the probability of each link to be observed, $q^{(2)}_a = vec(G(t))_a$, with $\theta^{(2)}_a = 2(h_a - 1)$; and the last group of statistics, $q^{(3)}_a = vec(G(t-1))_a$, is related with the presence or absence of each link at the previous time step with parameters $q^{(3)}_a = 2\sum_b J_{ab}$.

The DyNoKIM allows us to consider time varying θ_i and to estimate the forecast accuracy of the model at each time step, as showed with the link prediction example presented in the main text.

Interestingly, a wide range of TERGM specifications can be mapped to the KIM. As a simple example, let us consider a TERGM with two terms only

$$P(G(t)|G(t-1),\theta) = \frac{e^{\sum_{ij}[\theta_{dens} G_{ij}(t) + \theta_{stab}(G_{ij}(t) G_{ij}(t-1) + (1-G_{ij}(t))(1-G_{ij}(t-1)))]}}{K(\theta)}. \tag{18}$$

This can be rewritten as

$$P(s(t)|s(t-1),\theta) = \frac{e^{N\theta_{dens}/2 + \sum_k (s_k(t)\theta_{dens}/2 + s_k(t) s_k(t-1)\theta_{stab}/2)}}{K(\theta)},$$

which is exactly equivalent to a KIM restricted to have just two parameters $J_{ij} = J_{diag} = \theta_{stab}/2\ \forall i,j$ and $h_i = h_0 = \theta_{dens}/2\ \forall i$, and, absorbing the constants in the normalization function, we have

$$P(s(t)|s(t-1), J_{diag}, h_0) = \frac{e^{\sum_k s_k(t) h_0 + s_k(t) s_k(t) J_{diag}}}{Z(\theta)}.$$

If we consider the DyNoKIM extension of such a restricted KIM we obtain

$$P(s(t)|s(t-1), J_{diag}, h_0, \beta(t)) = \frac{e^{\beta(t)\sum_k (s_k(t) h_0 + s_k(t) s_k(t-1) J_{diag})}}{Z(\theta)} \tag{19}$$

that is effectively an extended version of the initial TERGM. Moreover, it is easy to see that the DyEnKIM results in the following

$$P(s(t)|s(t-1), J_{diag}, h_0, \beta(t)) = \frac{e^{\sum_k (s_k(t)\beta_h(t)h_0 + s_k(t)s_k(t-1)\beta_{diag}(t)J_{diag})}}{Z(\theta)}. \quad (20)$$

that maps to a version of (18) with dynamical parameters $\theta_{dens}(t)$ and $\theta_{stab}(t)$ evolving independently. We believe this observation is very relevant as it is an extension of the TERGM at hand to its version where each parameter is allowed to follow its own evolution, potentially unrelated to the others. This is a different evolution from the DyNoKIM's one, as the latter is driven by a single $\beta(t)$ and maps into comoving TERGM parameters. Indeed, also in this context, the two models have different purposes and different applicability. TERGM extensions resulting from DyNoKIM allow us to quantify forecast accuracy, similarly to what showed in the main text, while DyEnKIM, similarly to what we discussed in the applications presented and in numerical simulations, allows for a decoupling of the temporal relevance of different network statistics.

As a final remark, we point out that the class of TERGMs that can be mapped into KIMs, and benefit of the corresponding score driven extensions, is not restricted to cases with linear dependency on the lagged adjacency matrix. In fact, we can also consider network's statistics depending on products of lagged matrix elements, e.g. $h(G(t), G(t-1)) = \sum_{ijk} G_{ik}(t)G_{ij}(t-1)G_{ik}(t-1)$, as long as they depend linearly on $G(t)$. A TERGM with such statistics can be mapped in a KIM with the addition of predetermined regressors and is easily extended, for example, to the corresponding DyNoKIM version. For example, all the statistics discussed as explicit examples in [39] take this form, and can be mapped into a KIM. Although a full characterization of the set of TERGM's specifications that can be mapped into a KIM lies outside the scope of this work, we suspect it to be very large, and potentially include all statistics commonly used in practice.

In summary, as we have shown that KIM belongs to the TERGM family, and its score driven extensions result in extensions of the corresponding TERGM. That is the case for both DyNoKIM and DyEnKIM, as we showed explicitly for a simple TERGM specification. We believe that our findings open up a vast space of potential applications of score driven KIM to temporal networks, and raise interesting theoretical questions on the possibility of mapping a generic TERGM into a KIM, that we leave for future explorations.

Identifying traders strategy changes around macroeconomic news

As a second application of DyEnKIM we consider the problem of investigating the time-varying reaction of financial traders to the arrival of macroeconomic news, a typically non-stationary situation. This analysis can be seen as an extension to a time-varying parameter setting of a previous work by some of the authors [17], where the standard Kinetic Ising Model was used to infer a network of lead-lag relationships among traders strategies in the spot exchange rate market between the Euro and US Dollar. Here the time series represent buy ($s_i = +1$) or sell ($s_i = -1$) trades of Euro for US Dollars, performed by a set of traders $i = 1, \dots, N$ in the period May - September 2013 on the electronic

Foreign Exchange platform of a major dealer in the market, who provided the data. Time is discretized in 5 minutes time bins (see the Data paragraph below for further details). Since traders are likely to be influenced by price movements, we include the exchange rate log-returns in the previous time step as an external covariate in the DyEnKIM. This leads to an extended DyEnKIM where

$$\begin{aligned} g_i(t) &= \sum_j J_{ij}(t)s_j(t-1) + h_i(t) + b_i(t)r(t-1) \\ b_i(t) &= \beta_b(t)b_i. \end{aligned}$$

The coupling parameter b_i models trader i's average strategic behavior in response to past price changes, and an additional score-driven parameter $\beta_b(t)$ modulates the intensity of this effect on all traders. The definitions of $J_{ij}(t)$ and $h_i(t)$ are unchanged from Eq. 8 in the main text.

We split the data into monthly samples to account for traders entering and leaving the market over time. We then proceed to estimate the model on each monthly sample and filter the time-varying parameters $\boldsymbol{\beta}(t) = (\beta_{diag}(t), \beta_{off}(t), \beta_h(t), \beta_b(t))$ and h_0. As proposed in the original paper [17], this model should be interpreted as a way to put in relation the strategic decisions made by traders, highlighting which market participants can carry information about short-term trends in demand and supply as well as identifying the relations between traders adopting different strategies. In this extended score-driven version, the time-varying parameters allow a more refined interpretation of the model results by making explicit *when* the traders are more "coupled" with others or affected by price variations in their strategic behavior.

We expect that the publication of relevant news is likely to affect the behavior of traders, causing them to deviate from their "ordinary" trading strategies. Given the specification of the DyEnKIM then it is likely that such deviations are captured by the time-varying $\boldsymbol{\beta}(t)$ and h_0. We thus test this hypothesis on a dataset of scheduled macroeconomic announcements (e.g. interest rate decisions by central banks, quarterly unemployment rate reports, ...) related to the EUR or USD, which amounts to a total of 474 non-overlapping announcement events, 283 affecting the US Dollar and the remaining 201 regarding the Euro.

As we compare different models for different months, we need to standardize the time series of our time-varying parameters. We begin by performing a multiplicative trend-seasonal decomposition on the elements of $\boldsymbol{\beta}$ and an additive decomposition on h_0, i.e.

$$\begin{aligned} \beta_k(t) &= \beta_k^{seas}(t)\beta_k^{trend}(t)\beta_k^{rand}(t) \\ h_0(t) &= h_0^{seas}(t) + h_0^{trend}(t) + h_0^{rand}(t) \end{aligned}$$

where $k \in \{diag, off, h, b\}$. A Fourier analysis of the time series identifies the principal spectral component at daily frequency; we then set the seasonal component to have daily periodicity, capturing any intraday pattern the parameters might show, while the trend component is the moving average of the parameter through a two-side square filter with bandwidth of one day, to match the seasonality. The residual components β_k^{rand} and h_0^{rand} are then the variables of interest, as they are not explained by either the intraday pattern or the local average value.

In order to compare the residuals from different monthly samples, and given that quantile-quantile plots suggest that the residuals are close to being Gaussian distributed, we consider the associated z-scores $\hat{\beta}_k^{rand}(t)$ and $\hat{h}_0^{rand}(t)$; we then take, for each lag l in a two hours time window around the events, the average value of the z-scores across all events, that is

$$\langle \hat{\beta}_k^{rand}(l) \rangle = \frac{1}{N_e} \sum_{e=1}^{N_e} \hat{\beta}_k^{rand}(t_e^* + l)$$

where N_e is the number of macroeconomic news events, $l \in [-60m, 60m]$ and t_e^* is the time interval within which event e takes place, i.e. the announcement happens in the time window $(t_e^* - 5m, t_e^*]$.

Figure S9 shows the averaged z-scores for all the $\boldsymbol{\beta}$ components, along with 95% confidence intervals. We observe significant deviations from the confidence intervals in proximity of the news announcement. Specifically, both the β_{diag} and the β_{off} parameters indicate a reduction in the importance of both autocorrelation and cross-correlation of the trading behavior of traders, while the exogenous component β_h shows less significant effects. The β_b parameter is also marginally smaller in the minutes following the announcement, suggesting that traders are less focused on reacting to the price dynamics when news are released. Thus the analysis of time varying βs indicate that, in proximity of scheduled macroeconomic news, trading activity becomes less affected by other traders and by the behavior of the price.

We find the most significant effects on the z-scores of h_0^{rand}, shown in Figure S10. Given the processing of our data, $h_0 > 0$ means that the collective trading activity is more directed towards purchasing EUR in exchange for USD, and viceversa when $h_0 < 0$. Looking at the top panel of Figure S10 it might then seem that around news traders tend to buy more USD. However we selected news affecting either USD or EUR: the result changes significantly once we split the sample in USD-related and EUR-related news.

In the middle and bottom panels of Figure S10 we show the average pattern of $\hat{h}_0^{rand}$ around USD-affecting news and EUR-affecting news, respectively. What we find is that traders are actually more likely to drop the affected currency from their inventory when they know a news is coming, and that the effect is stronger for EUR than USD. We believe there are at least two plausible explanations for this: one is that the market we analyze is situated in London, thus the majority of the traders are likely to be European; another is that, being the data from 2013, the Euro debt crisis is affecting EUR-related news, causing more risk-aversion in traders.

We also argue that this particular behavior is expected by the set of selected traders: as we only consider agents that perform trades in at least 30% of the 5 minutes time windows, we are very likely excluding the ones that follow a "news-trading" strategy - i.e. based on prediction and reaction to news - who typically would not trade that often. This might explain why the selected traders show risk-aversion around news, selling the affected currency before the announcements to avoid adverse selection and higher volatility.

Data. We obtained data on EUR-USD trading from a major dealer in the foreign exchange market, who offers the trading platform as a service to its clients. Given the sensitive nature of the data these are not publicly available.

The trading records contain the time of trade with millisecond precision, the anonymous identifier of the trader, the volume $v_i(k)$ of EUR purchased for USD in transaction k by trader i (negative in case the trader sells EUR for USD) and the price paid. The data we use spans the period May - September 2013, in London market opening time (8AM - 4.30PM GMT, Monday to Friday). We split the data in monthly samples and we discretize time in 5 minutes time windows. For each time window t we consider the total traded volume by i, $V_i(t) = \sum_{k \in t} v_i(k)$, and take $s_i(t) = \text{sign}[V_i(t)]$, hence $s_i(t) = +1$ if trader i has purchased more EUR for USD in time window t and $s_i(t) = -1$ otherwise.

Not all the traders perform trades every 5 minutes, meaning that the data contains missing values. We select traders that are active in at least 30% of the 5 minutes time windows each month, reducing our sample to 15-20 traders depending on the month, and the missing values are filled by an Expectation-Maximization-like procedure, as shown by the same authors [16]. The rationale behind this choice is that, since the dataset only covers a fraction of the market (namely traders active on a specific trading platform of the many available), it is reasonable to assume that traders not trading on the platform (or other traders with similar strategies) could still be active on other venues, and thus being able to assign an expected value to a missing observation can be useful. Indeed the imputation of these missing trades has been proved to be financially significant to estimate causal effects between herding and liquidity in fragmented markets [17]. We refer the interested reader to the original papers [16, 17] for further methodological detail.

Macroeconomic news data collected from *www.dailyfx.com.* A timestamp is associated with each news release, with labels characterizing which currencies are affected and the level of importance (low, medium or high) of the news. We consider news that are labeled as highly important and involving either USD or EUR.

Disentangling endogenous and exogenous price dynamics

As a second application of the DyEnKIM we consider the problem of quantifying the contribution to stock price changes due to exogenous events (e.g. news, announcements) and to endogenous feedbacks. A vast literature [40, 41, 42, 43, 44] has tackled this point, but almost invariably this has been done by assuming that the relation between price and external drivers, as well as those driving the internal feedback, is constant in time. The DyEnKIM allows us to test this hypothesis, by considering time varying parameters whose dynamic can be filtered from data. Understanding the role of exogenous or endogenous drivers in market volatility is very important, also to devise possible policy measures able to avoid their occurrences and DyEnKIM, being able to identify them in real time, could provide valuable tools for market monitoring.

For this application we focus on two events that caused huge turmoil in the stock markets at the intraday level. The first one is the May 6, 2010 Flash Crash, when a seemingly unjustifiable sudden drop in the price of E-mini S&P 500 futures contracts caused all major stock indices to plummet in a matter of a few minutes, recovering most of the lost value when circuit breakers came into place. Multiple explanations of what happened have been offered by a large number of academics, regulators and practitioners: responsibility has been attributed to careless algorithmic trading [45], deteriorated market liquidity which

quickly vanished when price volatility increased [46], market fragmentation [47], predatory trading strategies by high-frequency traders [48, 49].

The second event we analyze is the announcement following the Federal Open Market Committee (FOMC) meeting of July 31, 2019. In this meeting the Federal Reserve operated its first interest rate cut in over a decade, the last one dating back to the 2008 financial crisis, encountering mixed reactions in both the news and the markets. In particular an answer to a question in the Q&A press conference by the Fed Chairman Powell has been highlighted by news agencies, when being asked whether further cuts in the future meetings were an option, he answered "we're thinking of it essentially as a midcycle adjustment to policy" [50]. This answer triggered turmoil in the equity markets, with all major indices dropping around 2% in a few minutes.

Like in the previous section, we construct our dataset for both events taking price movements for the then S&P100-indexed stocks at the 5 seconds time scale and constructing the associated price activity time series. Differently from the previous example, here we apply the DyEnKIM methodology to study variations in the relative importance of different sets of parameters as events unfold. In this case the Lagrange Multiplier test rejects the null of constant parameters for all βs and all datasets. To better interpret the results we introduce the value of the components of the effective fields $g_i(t)$, each related to one of the time-varying parameters

$$
\begin{aligned}
g_i(t) &= g_{i,diag}(t) + g_{i,off}(t) + g_{i,h}(t) \\
g_{i,diag}(t) &= \beta_{diag}(t) J_{ii} s_i(t) \\
g_{i,off}(t) &= \beta_{off}(t) \sum_j J_{ij} s_j(t) \\
g_{i,h}(t) &= \beta_h(t)(h_i + h_0(t))
\end{aligned}
$$

which we then average at each time across all indices i, obtaining the quantities $\langle g_{diag} \rangle(t)$ and so on.

Since the model is applied to price activity, which can be thought of as a proxy of high-frequency volatility [40, 41], the financial interpretation of these time-varying parameters relates to volatility clustering in the case of β_{diag}, to volatility spillovers for β_{off}, to higher or lower market-wise volatility for h_0 and the relevance of exogenous effects is given by β_h. Thus the $\langle g_\cdot \rangle(t)$ quantities can be intuitively related to what the explained sum of squares means for linear regression models, in the sense that the more a $\langle g_\cdot \rangle(t)$ is far from 0 relative to others the more the data are affected at time t by that subset of parameters and the corresponding variable. We choose to show these quantities as a simple way of assessing the relevance of the components, a problem that is not easily solved in this kind of models.

The top panel of Figure S11 shows the components of the fields during the Flash Crash of May 6, 2010. Here the parameters show a very significant variation around the crash, with a large increase of $\langle g_h \rangle$ in the 45 minutes preceding the crash together with a similar increase of the endogeneity field $\langle g_{diag} \rangle$ and $\langle g_{off} \rangle$ during the event, which then stay large until market close. This indicates that the turmoil induced by the Flash Crash reverberated for the remainder of the trading hours, even after the prices had recovered at pre-crash levels. The intraday pattern is overshadowed by the effect of the crash,

but the picture at the beginning of the day is similar to normal trading days (see Fig. S12). These results indicate an exogenous increase in activity before the crash, which is accompanied by the endogenous mechanism of volatility spillovers between stocks, as evidenced by large value of $\langle g_{off} \rangle$ during and after the Flash Crash. In conclusion our analysis indicates that both exogenous and endogenous drivers were important for the onset of Flash Crash.

In the bottom panel of Figure S11 we show the values of the effective fields on July 31, 2019. The FOMC announcement went public at 14:00:00 EST and is followed by a press conference at 14:30:00 EST, with a Q&A starting at around 14:36:00 EST. Again we see that the usual intraday pattern is interrupted by the news, which however, differently from the Flash Crash, was a scheduled event. This difference leads to the complete absence of any sort of "unusual" effect in the earlier hours of the day, as typically analysts provide forecasts regarding these announcements in the previous days and this information is already incorporated in the prices. What then happens is that, if the news does not meet market expectations, a correction in prices will occur as soon as the information is made public, leading to higher market volatility in the minutes and hours following the announcement [51]. In this specific case, forecasts were mixed between a 0.25% and a 0.50% interest rates cut scenario.

The published announcement at 14:00 EST mostly matched these forecasts, with the FOMC lowering the interest target rate by 0.25%, and we indeed see that the price levels are not particularly affected by the news. However a transient increase in volatility, and in particular the endogenous components, can still be observed in the few minutes following the announcement, quickly returning to average levels. It is interesting to see the reaction to the press conference held 30 minutes after the release, and in particular to the answers the Chairman of the Fed Jerome H. Powell gives to journalists in the Q&A. As soon as the Q&A starts, around 14:36 EST, prices begin to plummet in response to the Chairman's answers, possibly reacting to the statement that this interest rates cut was only intended as a "midcycle adjustment to policy" rather than as the first of a series. Expectations of further rates cuts in the later months of the year could be a reason for this adjustment in the prices when these forecasts are not met, as usually lower interest rates push the stock prices up. We see however that this unexpected event causes a behavior in the estimated time-varying parameters resembling what we have seen in the Flash Crash, albeit the endogenous components are even more significant here.

Table of acronyms

Acronym	Spelling
**KIM**	Kinetic Ising Model
**DyNoKIM**	Dynamical Noise Kinetic Ising Model
**DyEnKIM**	Dynamic Endogeneity Kinetic Ising Model
**GARCH**	Generalized AutoRegressive Conditional Heteroskedasticity (model)
**ROC**	Receiver Operating Characteristic (curve)
**AUC**	Area Under the (ROC) Curve
**SI**	Supplementary Information
**KS**	Kolmogorov-Smirnov (distance)
**ADAM**	ADAptive Momentum estimation
**TP (FP) / TN (FN)**	True (False) Positive / True (False) Negative
**TPR (FPR)**	True (False) Positives Ratio
**TERGM**	Temporal Exponential Random Graph Model

References

[1] Ernst Ising. Beitrag zur theorie des ferromagnetismus. Zeitschrift für Physik A Hadrons and Nuclei, 31(1):253–258, 1925.

[2] Lars Onsager. Crystal statistics. i. a two-dimensional model with an order-disorder transition. Physical Review, 65(3-4):117, 1944.

[3] Scott Kirkpatrick and David Sherrington. Infinite-ranged models of spin-glasses. Physical Review B, 17(11):4384, 1978.

[4] Seiji Tanaka and Harold A Scheraga. Model of protein folding: incorporation of a one-dimensional short-range (ising) model into a three-dimensional model. Proceedings of the National Academy of Sciences, 74(4):1320–1323, 1977.

[5] John J Hopfield. Neural networks and physical systems with emergent collective computational abilities. Proceedings of the national academy of sciences, 79(8):2554–2558, 1982.

[6] Jean-Philippe Bouchaud. Crises and collective socio-economic phenomena: Simple models and challenges. Journal of Statistical Physics, 151(3):567–606, May 2013.

[7] Edwin T Jaynes. Information theory and statistical mechanics. Physical review, 106(4):620, 1957.

[8] Claude E Shannon. A mathematical theory of communication. Bell system technical journal, 27(3):379–423, 1948.

[9] Bernard Derrida, Elizabeth Gardner, and Anne Zippelius. An exactly solvable asymmetric neural network model. EPL (Europhysics Letters), 4(2):167, 1987.

[10] A Crisanti and Haim Sompolinsky. Dynamics of spin systems with randomly asymmetric bonds: Ising spins and glauber dynamics. Physical Review A, 37(12):4865, 1988.

[11] ACC Coolen. Statistical mechanics of recurrent neural networks i: Statics. Handbook of biological physics, 4:531–596, 2001.

[12] ACC Coolen. Statistical mechanics of recurrent neural networks iidynamics. Handbook of biological physics, 4:619–684, 2001.

[13] Joanna Tyrcha, Yasser Roudi, Matteo Marsili, and John Hertz. The effect of nonstationarity on models inferred from neural data. Journal of Statistical Mechanics: Theory and Experiment, 2013(03):P03005, 2013.

[14] Benjamin Dunn and Yasser Roudi. Learning and inference in a nonequilibrium ising model with hidden nodes. Physical Review E, 87(2):022127, 2013.

[15] Aurélien Decelle and Pan Zhang. Inference of the sparse kinetic ising model using the decimation method. Physical Review E, 91(5):052136, 2015.

[16] Carlo Campajola, Fabrizio Lillo, and Daniele Tantari. Inference of the kinetic ising model with heterogeneous missing data. Physical Review E, 99(6):062138, 2019.

[17] Carlo Campajola, Fabrizio Lillo, and Daniele Tantari. Unveiling the relation between herding and liquidity with trader lead-lag networks. Quantitative Finance, 20(11):1765–1778, 2020.

[18] Patricia A Jacobs and Peter AW Lewis. Discrete time series generated by mixtures. III. autoregressive processes (DAR (p)). Technical report, Naval Postgraduate School Monterey Calif, 1978.

[19] Carlo Campajola, Fabrizio Lillo, Piero Mazzarisi, and Daniele Tantari. On the equivalence between the kinetic ising model and discrete autoregressive processes. Journal of Statistical Mechanics: Theory and Experiment, 2021(3):033412, 2021.

[20] Marco P Tucci. Time-varying parameters: a critical introduction. Structural Change and Economic Dynamics, 6(2):237–260, 1995.

[21] Siem Jan Koopman, Andre Lucas, and Marcel Scharth. Predicting time-varying parameters with parameter-driven and observation-driven models. Review of Economics and Statistics, 98(1):97–110, 2016.

[22] David R Cox, Gudmundur Gudmundsson, Georg Lindgren, Lennart Bondesson, Erik Harsaae, Petter Laake, Katarina Juselius, and Steffen L Lauritzen. Statistical analysis of time series: Some recent developments [with discussion and reply]. Scandinavian Journal of Statistics, pages 93–115, 1981.

[23] George E Tauchen and Mark Pitts. The price variability-volume relationship on speculative markets. Econometrica: Journal of the Econometric Society, pages 485–505, 1983.

[24] Neil Shephard. Stochastic volatility: selected readings. Oxford University Press on Demand, 2005.

[25] Luc Bauwens and David Veredas. The stochastic conditional duration model: a latent variable model for the analysis of financial durations. Journal of econometrics, 119(2):381–412, 2004.

[26] Christian M Hafner and Hans Manner. Dynamic stochastic copula models: Estimation, inference and applications. Journal of Applied Econometrics, 27(2):269–295, 2012.

[27] Tim Bollerslev. Generalized autoregressive conditional heteroskedasticity. Journal of econometrics, 31(3):307–327, 1986.

[28] Drew Creal, Siem Jan Koopman, and André Lucas. Generalized autoregressive score models with applications. Journal of Applied Econometrics, 28(5):777–795, 2013.

[29] Andrew C. Harvey. Dynamic Models for Volatility and Heavy Tails: With Applications to Financial and Economic Time Series. Econometric Society Monographs. Cambridge University Press, 2013.

[30] Francisco Blasques, Siem Jan Koopman, and Andre Lucas. Information-theoretic optimality of observation-driven time series models for continuous responses. Biometrika, 102(2):325–343, 2015.

[31] Francisco Blasques, Andre Lucas, and Andries van Vlodrop. Finite sample optimality of score-driven volatility models. Tinbergen Institute Discussion Paper, 17-111/III, 2017.

[32] Francesco Calvori, Drew Creal, Siem Jan Koopman, and André Lucas. Testing for parameter instability across different modeling frameworks. Journal of Financial Econometrics, 15(2):223–246, 2017.

[33] Daniel B Nelson. Filtering and forecasting with misspecified arch models i: Getting the right variance with the wrong model. Journal of econometrics, 52(1-2):61–90, 1992.

[34] Marc Mézard and J Sakellariou. Exact mean-field inference in asymmetric kinetic ising systems. Journal of Statistical Mechanics: Theory and Experiment, 2011(07):L07001, 2011.

[35] Diederik P Kingma and Jimmy Ba. Adam: A method for stochastic optimization. arXiv preprint arXiv:1412.6980, 2014.

[36] Christian Francq, Lajos Horvath, and Jean-Michel Zakoïan. Merits and drawbacks of variance targeting in garch models. Journal of Financial Econometrics, 9(4):619–656, 2011.

[37] Yasser Roudi and John Hertz. Mean field theory for nonequilibrium network reconstruction. Physical review letters, 106(4):048702, 2011.

[38] Gašper Tkačik, Olivier Marre, Dario Amodei, Elad Schneidman, William Bialek, and Michael J Berry II. Searching for collective behavior in a large network of sensory neurons. PLoS Comput Biol, 10(1):e1003408, 2014.

[39] Steve Hanneke, Wenjie Fu, and Eric P. Xing. Discrete temporal models of social networks. Electronic Journal of Statistics, 4(none):585 – 605, 2010.

[40] Vladimir Filimonov and Didier Sornette. Quantifying reflexivity in financial markets: Toward a prediction of flash crashes. Physical Review E, 85(5):056108, 2012.

[41] Stephen J Hardiman, Nicolas Bercot, and Jean-Philippe Bouchaud. Critical reflexivity in financial markets: a hawkes process analysis. The European Physical Journal B, 86(10):442, 2013.

[42] Spencer Wheatley, Alexander Wehrli, and Didier Sornette. The endo–exo problem in high frequency financial price fluctuations and rejecting criticality. Quantitative Finance, 19(7):1165–1178, 2019.

[43] Marcello Rambaldi, Paris Pennesi, and Fabrizio Lillo. Modeling foreign exchange market activity around macroeconomic news: Hawkes-process approach. Physical Review E, 91(1):012819, 2015.

[44] Marcello Rambaldi, Vladimir Filimonov, and Fabrizio Lillo. Detection of intensity bursts using hawkes processes: An application to high-frequency financial data. Physical Review E, 97(3):032318, 2018.

[45] US Securities & Exchange Commission and Commodity Futures Trading Commission. Findings regarding the market events of may 6, 2010. Washington DC, 2010.

[46] David Easley, Marcos M Lopez De Prado, and Maureen O'Hara. The microstructure of the "flash crash": flow toxicity, liquidity crashes, and the probability of informed trading. The Journal of Portfolio Management, 37(2):118–128, 2011.

[47] Albert J Menkveld and Bart Zhou Yueshen. The flash crash: A cautionary tale about highly fragmented markets. Management Science, 65(10):4470–4488, 2019.

[48] Andrei Kirilenko, Albert S Kyle, Mehrdad Samadi, and Tugkan Tuzun. The flash crash: High-frequency trading in an electronic market. The Journal of Finance, 72(3):967–998, 2017.

[49] Matteo Aquilina, Eric Budish, and Peter O'Neill. Quantifying the high-frequency trading "arms race": A simple new methodology and estimates. United Kingdom Financial Conduct Authority Occasional Paper, 2020.

[50] Jerome Powell. Transcript of chair powell's press conference. Federal Open Market Committee, July 31, 2019.

[51] Nikolaus Hautsch, Dieter Hess, and David Veredas. The impact of macroeconomic news on quote adjustments, noise, and informational volatility. Journal of Banking & Finance, 35(10):2733–2746, 2011.

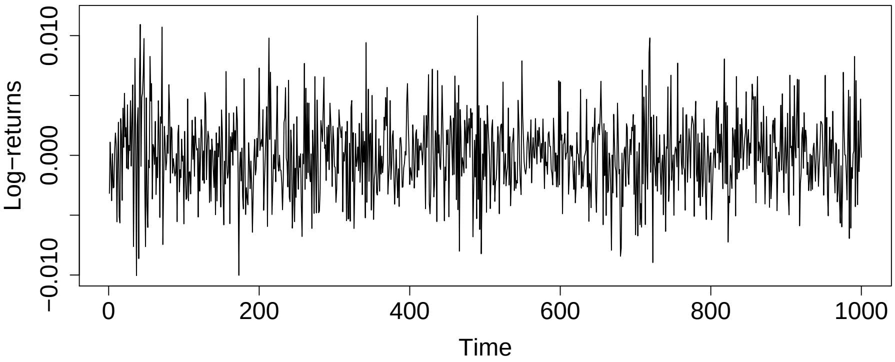

Figure S1: Artificially generated time series of length 1000 from a GARCH(1,1) model with $w = 10^{-6}$, $\alpha = 0.1$, $\beta = 0.8$.

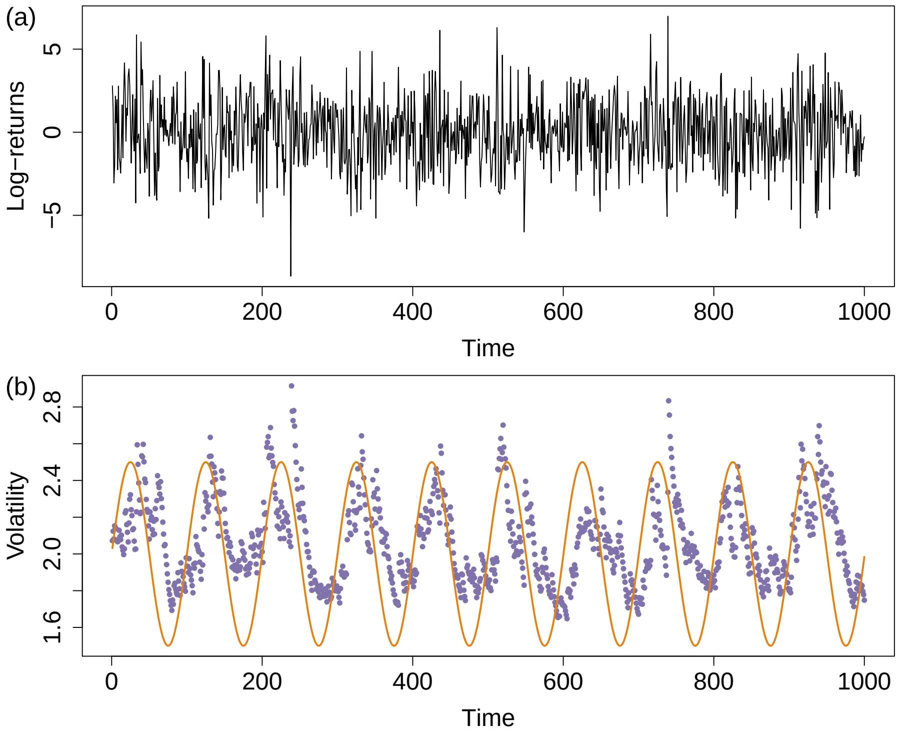

Figure S2: (a) Artificially generated time series of returns according to the model of Eq. 5. (b) Simulated (orange line) and filtered (purple dots) values of $\sigma(t)$. The latter are obtained by fitting a GARCH(1,1) model on the data in the left panel.

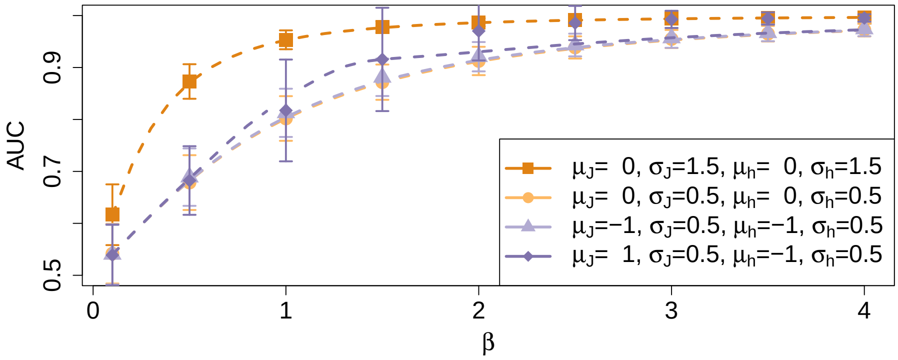

Figure S3: Comparison between the AUC estimated on data simulated from a Kinetic Ising Model and the theoretically derived AUC with Gaussian distribution of the J and h parameters, varying β and the hyperparameters μ_J, σ_J, μ_h and σ_h. Plot points report average simulated values for a given β with error bars at ± 1 standard deviation, dashed lines report theoretical values predicted by Eq. 8.

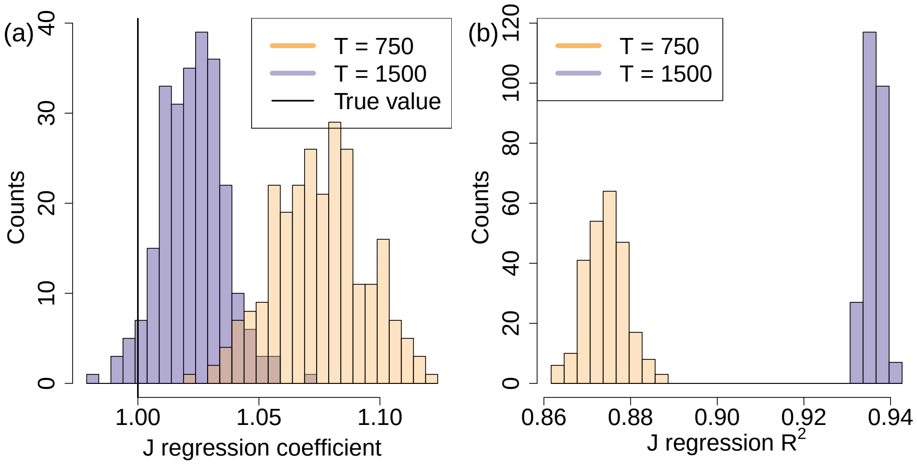

Figure S4: Consistency of the J matrix estimation. (a) Histogram of linear regression coefficients b between inferred and true values of J_{ij} over 250 samples for $N = 50$, $T = 750$ and $T = 1500$; (b) Histogram of coefficients of determination (R^2) for the same set of models. The convergence of both values towards 1 when increasing T is a sign of consistency of the estimation.

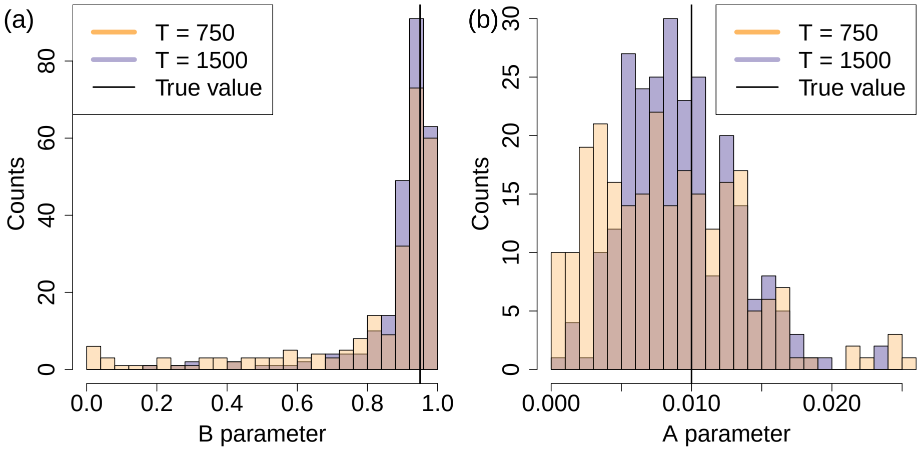

Figure S5: Consistency of the score-driven dynamics parameters. (a) Histogram of estimated values of B over 250 samples for $N = 50$, $T = 750$ and $T = 1500$; (b) Histogram of estimated values of A over 250 samples for the same set of models. The convergence towards the true value by increasing T is a sign of consistency of the estimation.

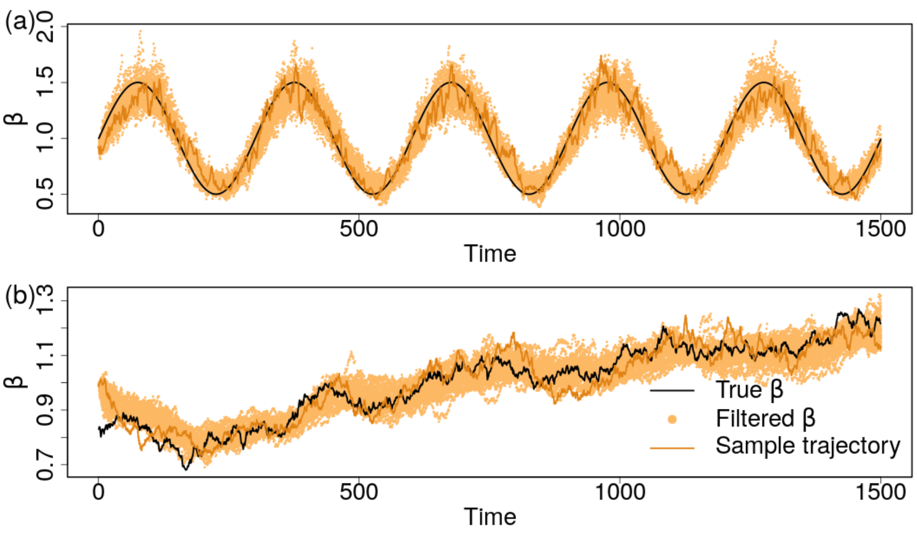

Figure S6: Simulation and estimation of a misspecified score-driven model over 30 simulations, with sample trajectories highlighted. (a) Deterministic β following a sinusoidal function; (b) Stochastic $\beta(t)$ following an AutoRegressive model of order 1.

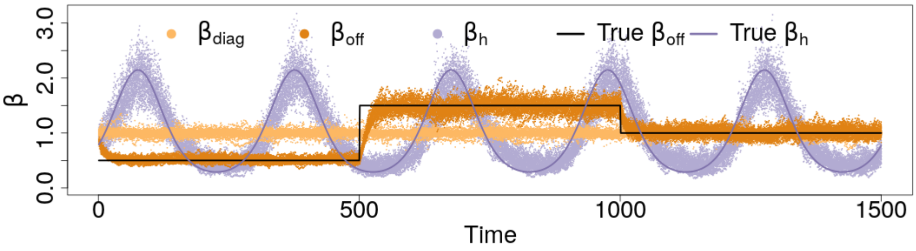

Figure S7: Estimation of $\beta_{diag}(t)$, $\beta_{off}(t)$ and $\beta_h(t)$ under model misspecification. The model was simulated with a constant $\beta_{diag}(t) = 1$, a piece-wise constant $\beta_{off}(t)$ and an exponentiated sinusoidal $\beta_h(t) = \frac{1}{\mathcal{J}_0(1)} \exp[\sin(\omega t)]$, with $\omega = 5\frac{2\pi}{T}$ and $\mathcal{J}_0$ the Bessel function of first kind of order 0 to normalize the mean. The points are the result of 30 different simulations and estimations, the lines show the values of β_{off} and β_h used to generate the data.

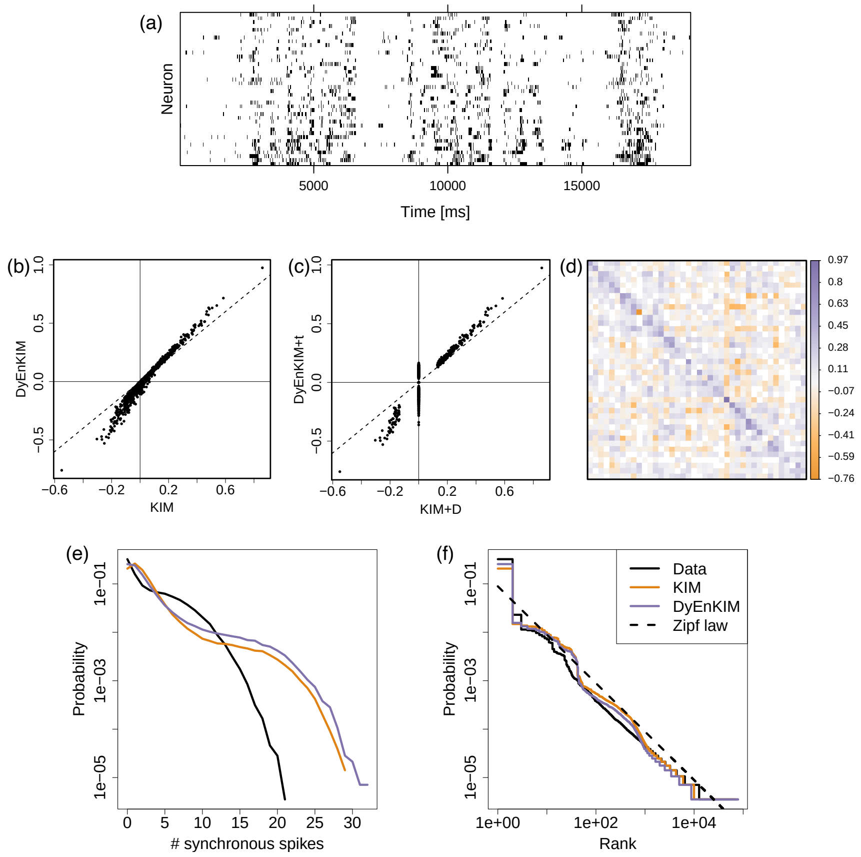

Figure S8: Additional results on the neuron spike data. (a) Raster plot for one sample in the data. Time is on the x-axis, neurons on the y-axis, a black dot at (t,i) indicates a spike from neuron i at time t; (b) Comparison between fitted values of J_{ij} using a KIM over the complete dataset (x-axis) and the average fitted values of J_{ij} with DyEnKIM correction, $\overline{J}_{ij} = \frac{1}{M}\sum_i^M J_{ij}^{(k)}$, where $J_{ij}^{(k)}$ is the J matrix fitted on sample k and M is the total number of experiments (y-axis). A dashed line is traced on the diagonal as guide to the eye; (c) The same as panel b after the Decimation pruning technique has been applied to the KIM and the t-test pruning has been applied to the DyEnKIM; (d) Visualization of the DyEnKIM $\overline{J}$ after the t-test pruning has been applied. White squares correspond to non-validated interactions; (e) Probability density function of the number of synchronous spikes. (f) Zipf plot of the frequency of observed patterns. In (e-f) the densities are obtained as average across the experiments, but a small variability is observed when considering individual experiments.

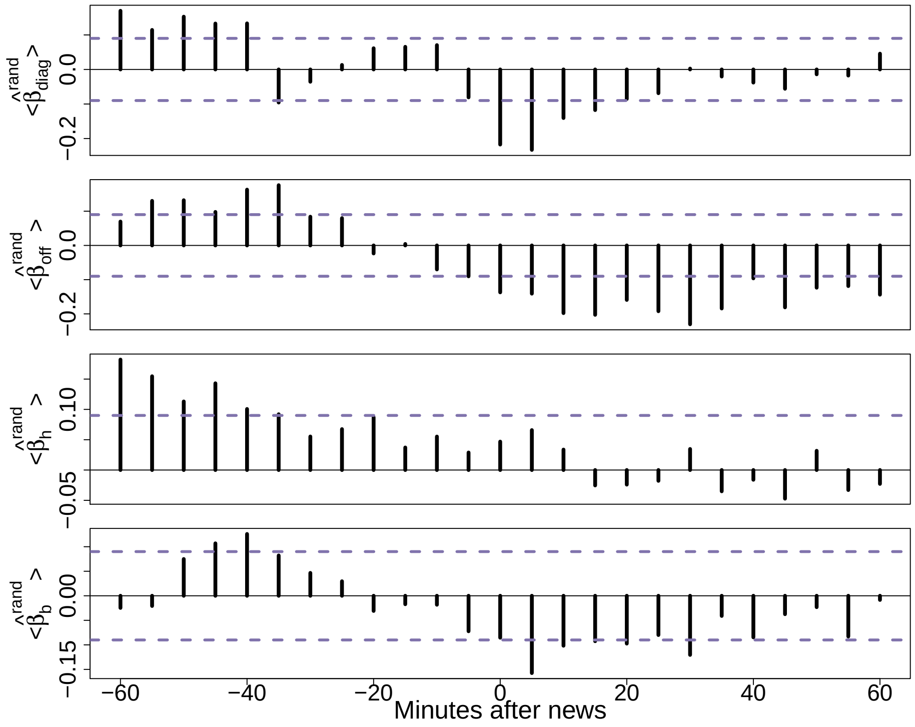

Figure S9: Patterns of the z-scores of $\boldsymbol{\beta}^{rand}(t)$ around macroeconomic news announcements, with purple dashed lines marking 95% confidence intervals for the null hypothesis of no variation with respect to the sample average.

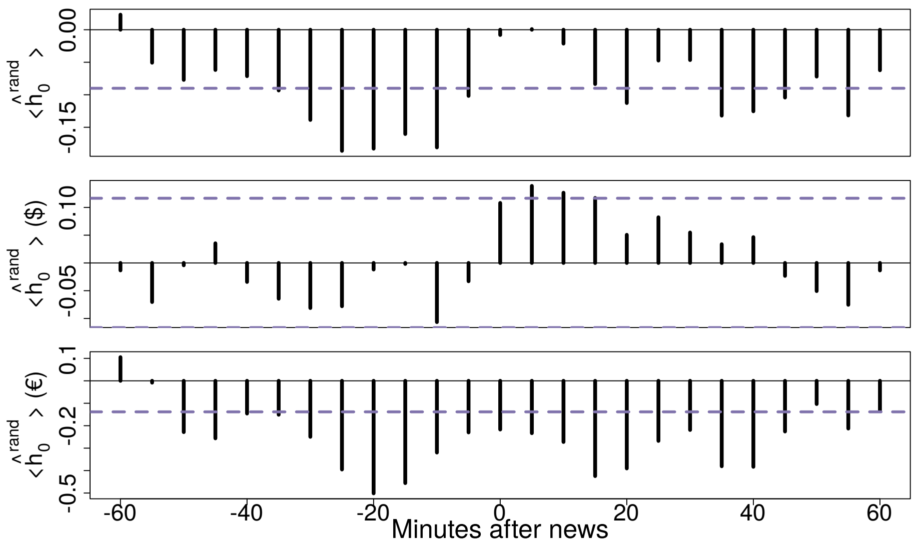

Figure S10: Patterns of the z-scores of $h_0^{rand}(t)$ around macroeconomic news, with purple dashed lines marking 95% confidence intervals for the null hypothesis of no variation with respect to the sample average. (top) all events; (mid) only USD-related events; (bottom) only EUR-related events.

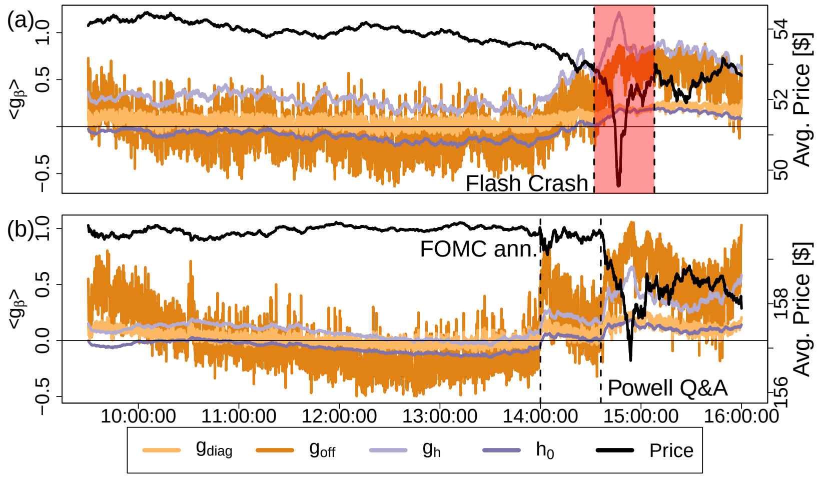

Figure S11: Values of $\langle g_{diag}\rangle(t)$, $\langle g_{off}\rangle(t)$, $\langle g_h\rangle(t)$ and $h_0(t)$ during the day of May 6, 2010 Flash Crash (a) and during the day of FOMC announcement on July 31, 2019 (b). The black lines are the the average midprice across the S&P100 stocks. The red area in the top panel highlights the time window (14:32:00 to 15:08:00 EST) where the Flash Crash takes place.

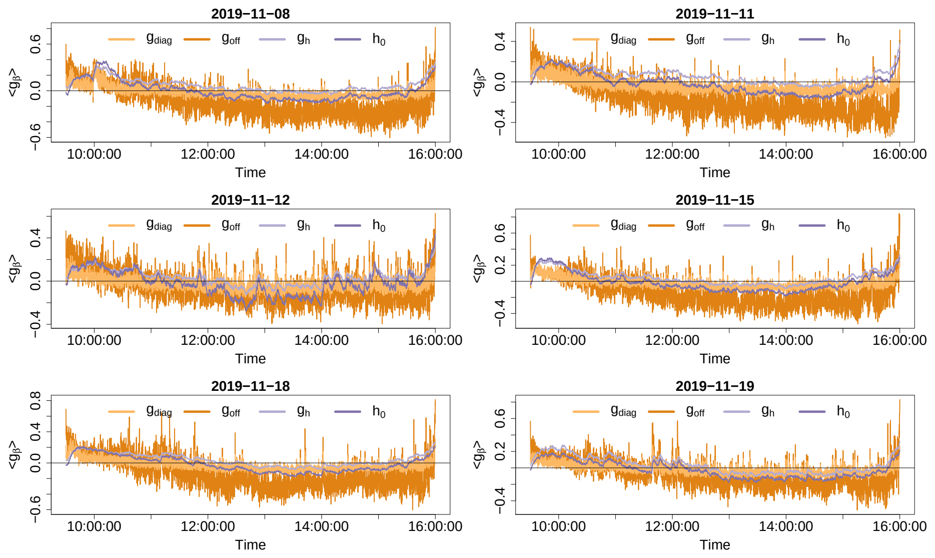

Figure S12: Values of $\langle g_{diag}\rangle(t)$, $\langle g_{off}\rangle(t)$, $\langle g_h\rangle(t)$ and $h_0(t)$ during six days in November 2019, when no abnormal event was recorded. The usual U-shaped pattern of intraday volatility and volume is observed.
